# Heavy metals in cigarette smoke strongly inhibit pancreatic ductal function and promote development of chronic pancreatitis

**DOI:** 10.1002/ctm2.1733

**Published:** 2024-06-14

**Authors:** Petra Pallagi, Emese Tóth, Marietta Görög, Viktória Venglovecz, Tamara Madácsy, Árpád Varga, Tünde Molnár, Noémi Papp, Viktória Szabó, Enikő Kúthy‐Sutus, Réka Molnár, Attila Ördög, Katalin Borka, Andrea Schnúr, Albert Kéri, Gyula Kajner, Kata Csekő, Emese Ritter, Dezső Csupor, Zsuzsanna Helyes, Gábor Galbács, Andrea Szentesi, László Czakó, Zoltán Rakonczay, Tamás Takács, József Maléth, Péter Hegyi

**Affiliations:** ^1^ Department of Medicine University of Szeged Szeged Hungary; ^2^ MTA–SZTE Momentum Epithelial Cell Signaling and Secretion Research Group, University of Szeged Szeged Hungary; ^3^ HCEMM–SZTE Molecular Gastroenterology Research Group, University of Szeged Szeged Hungary; ^4^ Department of Theoretical and Integrative Health Sciences University of Debrecen Szeged Hungary; ^5^ Translational Pancreatology Research Group, Interdisciplinary Centre of Excellence for Research Development and Innovation University of Szeged Szeged Hungary; ^6^ Department of Pharmacology and Pharmacotherapy University of Szeged Szeged Hungary; ^7^ Department of Plant Biology University of Szeged Szeged Hungary; ^8^ Department of Pathology Forensic and Insurance Medicine, Semmelweis University Budapest Hungary; ^9^ Department of Molecular and Analytical Chemistry University of Szeged Szeged Hungary; ^10^ Department of Pharmacology and Pharmacotherapy Medical School, University of Pécs Pécs Hungary; ^11^ National Laboratory of Drug Research and Development (Pharmalab) Budapest Hungary; ^12^ Institute of Pharmacognosy, Faculty of Pharmacy, University of Szeged Szeged Hungary; ^13^ Institute of Clinical Pharmacy, University of Szeged Szeged Hungary; ^14^ Institute for Translational Medicine, University of Pécs Pécs Hungary; ^15^ Eötvös Loránd Research Network Chronic Pain Research Group, University of Pécs Pécs Hungary; ^16^ Department of Pathophysiology University of Szeged Szeged Hungary; ^17^ Center of Translational Medicine and Institute of Pancreatic Disorders, Semmelweis University Budapest Hungary

**Keywords:** cadmium, CFTR, chronic pancreatitis, epithelial ion secretion, smoking

## Abstract

**Background and aims:**

Smoking is recognised as an independent risk factor in the development of chronic pancreatitis (CP). Cystic fibrosis transmembrane conductance regulator (CFTR) function and ductal fluid and bicarbonate secretion are also known to be impaired in CP, so it is crucial to understand the relationships between smoking, pancreatic ductal function and the development of CP.

**Methods:**

We measured sweat chloride (Cl^–^) concentrations in patients with and without CP, both smokers and non‐smokers, to assess CFTR activity. Serum heavy metal levels and tissue cadmium concentrations were determined by mass spectrometry in smoking and non‐smoking patients. Guinea pigs were exposed to cigarette smoke, and cigarette smoke extract (CSE) was prepared to characterise its effects on pancreatic HCO_3_^–^ and fluid secretion and CFTR function. We administered cerulein to both the smoking and non‐smoking groups of mice to induce pancreatitis.

**Results:**

Sweat samples from smokers, both with and without CP, exhibited elevated Cl^–^ concentrations compared to those from non‐smokers, indicating a decrease in CFTR activity due to smoking. Pancreatic tissues from smokers, regardless of CP status, displayed lower CFTR expression than those from non‐smokers. Serum levels of cadmium and mercury, as well as pancreatic tissue cadmium, were increased in smokers. Smoking, CSE, cadmium, mercury and nicotine all hindered fluid and HCO_3_^–^ secretion and CFTR activity in pancreatic ductal cells. These effects were mediated by sustained increases in intracellular calcium ([Ca^2+^]_i_), depletion of intracellular ATP (ATP_i_) and mitochondrial membrane depolarisation.

**Conclusion:**

Smoking impairs pancreatic ductal function and contributes to the development of CP. Heavy metals, notably cadmium, play a significant role in the harmful effects of smoking.

**Key points:**

1. Smoking and cigarette smoke extract diminish pancreatic ductal fluid and HCO_3_^–^ secretion as well as the expression and function of CFTR
2. Cd and Hg concentrations are significantly higher in the serum samples of smokers
3. Cd accumulates in the pancreatic tissue of smokers

## Graphical abstract

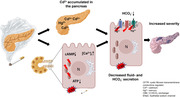

## 1 INTRODUCTION

Chronic pancreatitis (CP) is a multifactorial disease, in which factors that damage the pancreas impair the function of multiple cell types, resulting in severe tissue damage.^1^, ^2^, ^3^, ^4^, ^5^ The two most important risk factors for CP are alcohol and smoking,^6^, ^7^ with the pathophysiological mechanism of alcohol having been characterised quite well in recent years.^8^, ^9^, ^10^ It induces mitochondrial damage, endoplasmic reticulum (ER) stress and inhibition of secretion in pancreatic acinar cells,^11^ while it inhibits cystic fibrosis transmembrane conductance regulator (CFTR) activity and causes protein folding defects in pancreatic ductal cells.^12^ The toxic effects on these two cell types result in continuous cell death, activation of stellate cells^13^ and inflammation, leading to tissue atrophy, fibrosis and CP.^14^ It is important to note that not only alcohol has been characterised recently, but also its metabolites and biochemical end products (e.g., fatty acid ethyl ester).^15^, ^16^, ^17^, ^18^ However, much less is known about the effects of cigarette smoke constituents.

First and foremost, it should be noted that smoking (like alcohol consumption) causes dose‐dependent local tissue damage; in fact, it is clearly directly cytotoxic. Numerous studies have been published in the last few years on the mechanisms by which smoking damages acinar cells. Lugea et al. showed that cigarette smoke promotes cell death and features of pancreatitis in ethanol‐sensitised acinar cells by suppressing the adaptive unfolded protein response signalling pathway and activating the ER stress pathway.^10^ Wittel et al. showed that rats exposed to tobacco smoke developed pancreatic damage characterised by inflammation and reduced acinar cell number, similar to the features of CP.^19^ Moreover, tobacco smoke can alter gene expression, thus affecting the ratio of trypsinogen and serine protease inhibitor Kazal type 1 (SPINK1), which increases susceptibility to pancreatitis.^20^ There are conflicting data available on the effect of cigarette smoke on CFTR in epithelial cells. On the one hand, cigarette smoke has been shown to activate CFTR through reactive oxygen species‐stimulated cAMP signalling in human bronchial epithelial cells.^21^, ^22^ In contrast, cigarette smoke extract (CSE) has also been demonstrated to induce voltage‐dependent inhibition of CFTR expressed in *Xenopus* oocytes.^23^ Studies on human subjects have shown that past and current smokers have decreased pancreatic HCO_3_^−^ secretion, suggesting a dysfunction of the ductal epithelial cells when compared to never smokers.^24^, ^25^ Therefore, the aim of this study was to understand the effects and mechanism of smoking, one of the most important risk factors for CP, on pancreatic ductal epithelial cells (PDECs).

## 2 MATERIALS AND METHODS

### 2.1 Solutions and chemicals

Table S2 summarises the composition of the solutions used in fluorescent measurements. The pH of HEPES‐buffered solutions was set to 7.4 with HCl, whereas the HCO_3_^−^‐buffered solutions were gassed with 95% O_2_/5% CO_2_ to set pH. For patch clamp studies, the standard extracellular solution contained (in mmol/L): 145 NaCl, 4.5 KCl, 2 CaCl_2_, 1 MgCl_2_, 10 HEPES and 5 glucose (pH 7.4 adjusted with NaOH). The osmolarity of the external patch clamp solutions was 300 mOsm/L. The standard pipette solution contained (in mM): 120 CsCl, 2 MgCl_2_, .2 ethylene glycol‐bis(b‐aminoethyl ether)‐N,N,N8,N8‐tetraacetic acid, 10 HEPES and 1 Na_2_ATP (pH 7.2). 2.7‐Bis‐(2‐carboxyethyl)−5‐(and‐6‐)carboxyfluorescein‐acetoxymethylester (BCECF‐AM), 2‐(6‐(bis(carboxymethyl)amino)−5‐(2‐(2‐(bis(carboxymethyl)amino)−5‐methylphenoxy)ethoxy)−2‐benzofuranyl)−5‐oxazolecarboxylic‐acetoxymethylester (Fura2‐AM), MagnesiumGreen‐AM, Sodium‐binding benzofuran isophthalate acetoxymethylester (SBFI‐AM) and tetramethylrhodamine‐methylester (TMRM) were obtained from Invitrogen. Forskolin was purchased from Tocris. All other chemicals were obtained from Sigma–Aldrich, unless stated otherwise.

### 2.2 Human studies

#### 2.2.1 Ethics

The scheme of the experiments complied with the ethics of research and was approved by the Medical Research Council of Hungary under ETT TUKEB 24169‐2/2018/EKU licence number. It agrees with the declaration of the Medical World Federation proclaimed in Helsinki in 1964. Before enrolment, each volunteer/patient was given a detailed explanation of the nature, possible consequences and side effects of the study by a physician, after which they signed a written informed consent.

### 2.3 Characteristics of volunteers and patients

Patients admitted to the Pancreas Unit of the Department of Medicine, University of Pécs and Department of Medicine, University of Szeged were recruited for this study according to the following criteria. Table 1 describes the demographic details of the participants. Group 1: non‐smoker without CP (male:female ratio: 7:9, age 57 ± 4.1 years); group 2: smoker without CP (male:female ratio: 10:10, age 49.2 ± 2 years); group 3: non‐smoker with CP (male:female ratio: 8:4, age 61.6 ± 4.46 years); and group 4: smoker with CP (male:female ratio: 16:3, age 53 ± 3 years). A diagnosis of CP was based on the presence of at least two of the following criteria: constant or recurrent abdominal pain, characteristic findings in the pancreas confirmed by ultrasound, computed tomography or magnetic resonance imaging, ductal irregularities on endoscopic retrograde cholangiopancreatography or MR‐cholangiopancreatography examination, endoscopic ultrasound‐based diagnosis of CP and histologically confirmed CP.^1^ The smokers smoked at least half a pack of cigarettes a day for 1 year. Pilocarpine iontophoresis was conducted to collect sweat samples according to the method developed by Gibson and Cooke.^26^ Sweat chloride concentration (Cl_sw_^−^) was determined by conductance measurement using Wescor Sweat ChekTM 3100. Five milliliters of blood was drawn into a native yellow tube at the same time than the sweat test. After coagulation, the samples were centrifuged (3000 RCF, 15 min, 4°C) and stored at −20°C. Serum nicotine and heavy metal levels were measured.

**TABLE 1:** Demographic details of the participants.

	Group 1	Group 2	Group 3	Group 4
*N*	16	20	12	19
Average age	57.0	49.2	58.4	53.4
SD (age)	16.5	8.9	15.5	13.7
Male (*n*, %)	7 (44%)	10 (50%)	8 (67%)	16 (84%)
Female (*n*, %)	9 (56%)	10 (50%)	4 (33%)	3 (16%)
Average BMI	26.1	29.2	24.4	22.4
SD (BMI)	5.0	5.7	4.1	3.6
Current smokers (*n*, %)	0 (0%)	20 (100%)	0 (0%)	19 (100%)
Current smoker average packyears	NA	30,0	NA	20.4
SD (current packyear)	NA	25.0	NA	11.8
Former smokers (*n*, %)	9 (56%)	NA	7 (58%)	NA
Former smoker average packyear	5.5	NA	27.6	NA
SD (former packyear)	4.8	NA	22.7	NA

Abbreviations: BMI, body mass index; SD, standard deviation.

### 2.4 Detection of serum heavy metal content

An amount of 500 µL of nitric acid (65%, w/v) was added to 500 µL plasma samples to determine the elemental composition of serum samples. After 24 h of incubation, the samples were destroyed at 200°C and 1600 W for 15 min in a microwave‐assisted digestor (Mars Xpress 5). After appropriate dilution with double distilled H_2_O to the final volume of 5 mL, elemental concentrations were determined by inductively coupled plasma mass spectrometry (ICP‐MS; X‐Series 2, Thermo Scientific).

### 2.5 Human samples

Control pancreatic tissue samples (*n* = 5) were obtained from cadaver organ donors, who had no documented pancreatic disease (ethical approval no. 37/2017‐SZTE). Human pancreatic tissue samples from CP patients (*n* = 5) were obtained from surgical resections from pancreatic surgery.

### 2.6 Human and guinea pig organoid cultures

Human pancreatic organoid cultures were generated from cadaver donor‐derived pancreatic tissue samples (ethical approval no. 37/2017‐SZTE). Guinea pig and human pancreas tissues were cut into small pieces and the tissues were incubated in digestion media (Table S4) at 37°C in a vertical shaker for approximately 30 min. After the digestion, cells were collected by centrifugation in a 15 mL centrifuge tube (750 rpm, 10 min, 4°C) and washed twice by wash media (Table S5). The pellet was resuspended in wash media and Matrigel at a ratio of 1:5. About 10 µL of suspension was placed in one well of a 24‐well cell culture plate. After solidification, 500 µL of feeding media (Table S6) was applied in each well containing a dome and was changed every other day. The domes were centrifuged to pool and collect the cells for passaging. To remove Matrigel and separate the cells the domes were simultaneously worked by TrypLE Express Enzyme (Gibco, 12605028) at 37°C for 15 min in a vertical shaker followed by a washing step. Organoids were used for experiments between passage numbers 1−5. The composition of different media is listed in Tables S3–S6.

### 2.7 CAPAN‐1 cell cultures

The CAPAN‐1 cell line was purchased from the American Type Culture Collection and used for experiments between 20 and 60 passages. The cells were cultured according to the distributors’ instructions. For cAMP measurements, 2 × 10^5^ cells were seeded onto six‐well plates. Capan‐1 cells were cultured in Roswell Park Memorial Institute 1640 medium supplemented with 15% (v/v) foetal bovine serum, 1% (v/v) L‐glutamine and 1% (v/v) penicillin/streptomycin in a humidified incubator containing 5% CO_2_ at 37°C. Cell confluence was checked by light microscopy and the transepithelial electrical resistance was determined by using EVOM‐G Volt‐Ohm‐Meter (World Precision Instruments). Experiments were performed after the transepithelial electrical resistance of the monolayer increased to at least 50 Ω cm^2^.

### 2.8 Immunohistochemistry

Paraffin‐embedded, 3–4 µm thick sections of surgically removed resection specimens and control pancreas samples were used for immunohistochemistry. Immunohistochemical labelling was performed with a Leica Bond‐MAX Fully Automated IHC and ISH Staining System (Leica Biosystems). Briefly, deparaffinisation step was carried out with Bond Dewax Solution at 72°C. After washing with Bond Wash Solution, epitope retrieval was carried out with Bond Epitope Retrieval Solution 2 at 100°C for 20 min at pH 9. After slides were washed with wash solution at 35°C, peroxidase blocking was performed by Novocastra Peroxidase Block for 5 min. Primary antibody (anti‐CFTR antibody, Abcam, 1:400 dilution) were diluted in Bond primary antibody diluent and incubated on samples for 20 min. For immunohistochemical labelling and visualisation, Bond Polymer Refine Detection was applied for 8 min. For calculation of relative optical (RO) density, ImageJ program (National Institutes of Health) was used. Pixel values (PVs) were normalised to erythrocyte density in all cases. RO density value was calculated from the RO density = log_10_(255/PV_Norm_) equation as described earlier, assuming that the brightest value in the image was 255.

### 2.9 Tissue cadmium content

Lung and pancreas tissue samples were freeze‐dried for 24 h to remove water content before measuring dry weight. Dried tissues were digested using a closed vessel Anton Paar Multiwave 3000 microwave sample preparation system equipped with high‐purity modified polytetrafluoroethylene‐(PTFE‐TFM) vessels and infrared temperature monitoring. The operating conditions used during sample digestion are shown in Table S7. For digestion, ultra‐trace quality acids (Ultrace, VWR Chemicals) were used. The resulting clean solutions were diluted to 50 mL in volumetric flasks before analysis.

A quadrupole Agilent 7700X ICP‐MS was used for the quantitative trace analysis of cadmium (Cd). The sample introduction system consisted of an Agilent I‐AS autosampler and a Micro Mist pneumatic nebuliser equipped with a Peltier‐cooled Scott‐type spray chamber. The sample uptake rate was 600 µL/min. ICP plasma and interface parameters were set up as follows: RF forward power: 1550 W, plasma gas flow rate: 15.0 L/min, carrier gas flow rate: 1.05 L/min, and sampling depth: 10.0 mm. In order to avoid possible spectral interferences from the matrix of the samples, the measurements were performed in He mode using the Agilent ORS^3^ collision cell. Measurements were carried out by monitoring the signal of the ^111^Cd isotopes. ^115^In was used as an internal standard for Cd. In all ICP‐MS experiments, trace‐quality deionised lab water (Merck MilliPore Elix 10 equipped with a Synergy polishing unit) was used for the preparation of solutions. ICP‐MS tuning was performed on a daily basis using tuning solutions supplied by Agilent (no. G1820‐60410). Multi‐point, matrix‐matched calibration was executed using Inorganic Ventures IV‐ICPMS‐71A standard stock solution. The 99.996% purity argon and 99.999% purity helium gas used were purchased from Messer Hungarogáz. ICP‐MS data processing was performed with the Agilent Mass Hunter software.

### 2.10 Reverse transcription and quantitative real‐time polymerase chain reaction

CFTR mRNA expression of human pancreatic tissue was investigated using 3D organoid cultures derived from tissue samples of cadaver donors. To mimic the effects of smoking, organoid cultures were incubated for 30 min with 80 µg/mL CSE. Total RNA was isolated from tissue applying Macherey‐Nagel Nucleo Spin RNA Plus (Macherey‐Nagel). One microgram of total RNA from each sample was submitted to reverse transcription applying iScript cDNA Synthesis kit (Bio‐Rad) according to the manufacturer's protocol. Real‐time polymerase chain reactions were performed by ABI PRISM 7000 (Applied Biosystems) using triplicates of 100 ng cDNA and SsoAdvanced Universal SYBR Green Supermix (Bio‐Rad). Relative gene expression was calculated by the 2^ddCT^ method. The changes in mRNA expression level was calculated by normalising the threshold values to PolII.

### 2.11 Animal studies

#### 2.11.1 Ethics

The study was approved by the Hungarian National Scientific Ethical Committee on Animal Experimentation (licence no. XII./2222/2018).

### 2.12 Animals and pancreatic ductal fragments and cell isolation

Guinea pigs and FVB/N mice were kept at a constant room temperature (RT) of 24°C with a 12 h light–dark cycle and were allowed free access to standard chow and water in the Animal Facility of the Department of Medicine, University of Szeged. The gender ratio was 1:1 in both types of animals. Guinea pigs of age 4−8 weeks were humanly sacrificed by cervical dislocation. Pancreas was quickly removed and injected with isolation solution containing 100 U/mL collagenase (Worthington), .1 mg/mL trypsin inhibitor and 1 mg/mL bovine serum albumin (BSA), and intra/interlobular ducts were isolated by microdissection as described previously.^27^ Isolated ducts were then cultured overnight in a 37°C incubator gassed with 5% CO_2_/95% air as previously described.^27^ In some experiments, cultured ducts were incubated for 50 min at 37°C in 50 U/mL elastase dissolved in storage solution containing .1 mg/mL trypsin inhibitor and 30 mg/mL bovine serum albumin to obtain single pancreatic ductal cells.^27^ Then, the ducts were transferred into the cell isolation medium containing 145 mM NaCl, 4.5 mM KCl, 10 mM HEPES acid, 2 mM ethylene glycol‐bis(b‐aminoethyl ether)‐N,N,N8,N8‐tetraacetic acid and 10 mM glucose and incubated for further 10 min at 37°C. After the incubation, the ducts were transferred to a coverslip (24 mm) forming the base of a perfusion chamber and teased apart using stainless steel needles. The single pancreatic duct cells were then incubated in a storage solution for at least 30 min to allow the cells to attach to the coverslip. Following this, the cells were washed continuously with solutions at a rate of 3–4 mL/min and used within 3–4 h after isolation.

### 2.13 Preparation of cigarette smoke extract

CSE was used to test the acute effects of cigarette smoke on pancreatic ductal functions. CSE was prepared at the Department of Pharmacognosy, University of Szeged. Ten milliliters of deionised water (Milli‐Q, Merck) was aliquoted into a 50 mL test tube. The tube was closed up with a tee, with the tip of the tee reaching at least one‐half into the water. The resulting unit was connected to a Büchi V‐700 vacuum pump and 1 Kentucky Research Cigarette (3R4F; 12 mg tar and 1.0 mg nicotine) was inserted tightly into the other inlet. The cigarette was then lit and smoked continuously with the pump (950 mbar), and the smoke thus bubbled through the medium. This procedure was repeated with 15 cigarettes with the same medium. Dry weight was measured after evaporation of the crude extract with N2. CSE solution was then diluted to the appropriate concentration using HEPES or culture media. CSE was freshly prepared for each experiment or used within 2 days of preparation.

### 2.14 Exposure of guinea pigs to smoke inhalation

Guinea pigs were exposed to 1R3F research cigarette smoke (College of Agriculture, University of Kentucky) with the help of a manual smoking system (TE2; Teague Enterprises). Whole‐body smoke exposure was performed four times a day for 30 min in a closed chamber (two cigarettes were smoked per occasion for six guinea pigs) for a period of 6 weeks.^28^ Cigarettes were burned within 10 min; then, the ventilator was switched off for 30 min, and the chamber was ventilated afterward for 30 min. The average concentration of the total suspended particles in the chamber was ∼70 mg/m^3^. After the treatment period, the guinea pigs were sacrificed.

### 2.15 Measurement of pancreatic fluid secretion in vivo

Mice were exposed to cigarette smoke inhalation for 6 weeks as described above, and after the treatment period, pancreatic fluid was collected in vivo. Mice were anaesthetised with 125 mg/bw kg ketamine/12.5 mg/bw kg xylazine i.p. and placed on a heated pad to maintain body temperature. After median laparotomy, 4 mm diameter needle connected to an infusion catheter was introduced into the common biliopancreatic duct across the duodenum and the bile duct was occluded with a microvessel clip. After 30 min of secretin stimulation (75 CU/kg i.p.), the pancreatic juices was collected and the secretory rate was calculated as µL/bw g for 1 h.

### 2.16 Measurement of pancreatic fluid secretion in vitro

Swelling technique was used to investigate in vitro fluid secretion into the closed luminal space of the cultured guinea pig pancreatic ducts as described by Pallagi et al.^29^ Briefly, the ducts were attached to a poly‐L‐lysine precoated coverslip and transferred to a perfusion chamber (.45 mL). Bright‐field images were acquired at 1 min intervals using a CCD camera (CFW 1308C, Scion Corporation). At the end of each experiment, hypotonic solution was used to test the integrity of the duct wall. The analysis of the digital images of the ducts was performed with Scion Image software (Scion Corporation).

### 2.17 in vitro measurement of pH_i_, [Na^+^]_i_, [Ca^2+^]_i_, (ATP)_i_ and (∆Ψ)_m_

Isolated guinea pig pancreatic ducts were incubated with BCECF‐AM (1.5 µmol/L), SBFI (10 µmol/L), Fura2‐AM (2.5 µmol/L), MgGreen‐AM (5 µmol/L) or TMRM (100 nmol/L) for 30 min at 37°C in standard HEPES solution. The cover glasses were then transferred to a perfusion chamber mounted on an IX71 inverted microscope (Unicam). The measurements were carried out as described previously.^12^ HCO_3_^−^ efflux across the luminal membrane was determined two different, but complementary methods, as described previously.^29^ Briefly, luminal Cl^−^ removal technique was used to measure apical Cl^−^/HCO_3_^−^ exchange activity. pH_i_ elevation induced by removal of Cl^−^ from the luminal solution reflects the basolateral pNBC1 and apical Cl^−^/HCO_3_^−^ exchangers (CBE) activity, whereas re‐addition of Cl^−^ causes a decrease in pH_i_ because of secretion of HCO_3_^−^ via the CBE and the CFTR Cl^−^ channel. The rate of pH_i_ decrease (acidification) after luminal Cl^−^ re‐addition was calculated by linear regression analysis of pH_i_ measurements made over the first 30 s after exposure to the Cl^−^‐containing solution. In the other series of experiments cells were perfused with 20 mM NH_4_Cl in HCO_3_^−^/CO_2_‐buffered extracellular solution, which caused a rapid pH_i_ elevation due to the rapid influx of NH_3_ across the membrane. Recovery from alkalosis to basal pH_i_ value depends on the HCO_3_^−^ efflux (i.e., secretion) from the duct cells via Cl^−^/HCO_3_^−^ anion exchangers and CFTR. The first 30 s from the initial rate of recovery were analysed.

### 2.18 Electrophysiology

Single PDECs were prepared as described above. One drop of cell suspension was placed into a perfusion chamber mounted on an inverted microscope (Unicam) and allowed to settle for 30 min. Borosilicate glass capillaries (Clark) were used to pull patch clamp micropipettes by using a P‐97 Flaming/Brown micropipette puller (Sutter Co.) and their resistances were between 2.5 and 4 MΩ. To record membrane currents, whole‐cell configuration of patch clamp techniques was used with an EPC‐10 amplifier (Heka). After establishing a high‐resistance seal (1−10 GΩ) by gentle suction, the cell membrane beneath the tip of the pipette was disrupted. The series resistance was typically 4−8 MΩ before compensation (50%−80%, depending on the voltage protocol). Steady‐state current–voltage (*I*/*V*) relationships were obtained by holding Vm at 0 mV and clamping to ±100 mV in 20 mV increments. The membrane currents were digitised by using a 333‐kHz analogue‐to‐digital converter (Digidata1200; Axon Instruments) under software control (pClamp6; Axon Instruments). Analyses were performed by using Patchmaster software.

### 2.19 Cyclic AMP enzyme‐linked immunosorbent assay

To evaluate the molecular mechanism underlying the impact of smoke on ductal HCO_3_^–^ secretion and CFTR activity, cAMP production of Capan‐1 cells was determined using the direct cAMP enzyme‐linked immunosorbent assay (ELISA) kit (Enzo Life Sciences, ADI‐900‐066). 2 × 10^5^ Capan‐1 cells per well were seeded in six‐well culture plates for 24 h. After 24 h, the cells were treated with 20‒40‒80 µg/mL CSE or left untreated for 30 min. Briefly, the media was removed at the end of the treatment and the cells were lysed with 200 µL of . 1 M HCl for 10 min at RT. The lysates were centrifuged at 600 *g*, and the supernatants were transferred to clean tubes. The cAMP ELISA measurements were carried out following the protocol of the non‐acylated assay format, as described in the kit manual. The optical density of the samples was detected at 405 nm, using a FLUOstar OPTIMA microplate reader (BMG Labtech).

### 2.20 Immunofluorescent staining

Isolated pancreatic ducts were frozen in Shandon Cryomatrix and 5 µm thick sections were cut. Sections were fixed in 4% paraformaldehyde solution for 15 min. After washing in 1× Tris‐buffered saline (TBS), slides were stored in .1% goat serum and 10% BSA in TBS for non‐specific antigen blocking. Primary antibody (anti‐CFTR antibody, Abcam) was applied at a dilution of 1:100 in 1% BSA TBS for overnight at 4°C and then secondary anti‐rabbit antibody (Alexa Fluor 488; host: goat; Invitrogen Eugene) was used in a dilution of 1:400 for 2 h at RT. Nuclear staining was performed with 1 µg/mL Hoechst33342 for 15 min and sections were mounted with Flouromount. Images were captured with a Zeiss LSM880 confocal microscope using a 40× oil immersion objective (Zeiss, NA: 1.4).

### 2.21 Western blot

Guinea pig organoid cultures (GPOCs) of 24 Matrigel domes were treated with 80 µg/mL CSE for 12 h in a humidified 37°C incubator (5% CO_2_). Fresh feeding medium was changed on control GPOCs. After 12 h, Matrigel domes were collected and centrifuged (200 RCF, 10 min, 4°C, 180 mm rotor radius). Using TrypLE Express Enzyme at 37°C for 15 min in a horizontal shaker, Matrigel removal and cell separation were done concurrently, after which the cells were washed once. Cells were lysed in radioimmunoprecipitation assay (RIPA) lysis buffer containing complete protease inhibitor cocktail (Roche, 04693116001). Protein concentrations were determined by bicinchoninic acid (BCA).assay (Thermo Scientific, 23225). Then, 5× Laemmli buffer was added to the diluted samples and incubated for 30 min at 40°C. Samples were set on ice and 10 µg of protein was loaded onto an 8% Bis–Tris gel and transferred to a polyvinylidene difluoride (PVDF) (Thermo Scientific, 88520) membrane using the Bio‐Rad PROTEAN system (Bio‐Rad). The membranes were cut into four pieces and then blocked in blocking buffer (5% non‐fat milk + .1% Tween20 in TBS; TBS‐T) for 1 h at 4°C. First, proteins were detected by probing with anti‐beta‐actin (Cell Signaling Technology, 4967S, 1:5000) and anti‐CFTR (Alomone labs, ACL‐006, 1:500) overnight and with anti‐GAPDH (Cell Signaling Technology, 5174S, 1:10 000) for 30 min followed by anti‐rabbit horseradish peroxidase (HRP) (Invitrogen, 31460, 1:10 000) for 1 h. Protein signal was developed with Clarity Western ECL Substrate before being visualised on the ChemiDoc Imaging System (Bio‐Rad) (the exposition time was 1 min). Image colours were inverted to negative and the intensity of each lane was analysed in Fiji ImageJ (build 1.53c Java version 1.8.0_172, National Institutes of Health). Background intensity of the membrane was subtracted from the individual integrated density (intensity) values, normalised to GAPDH intensity and represented as ‘CFTR protein expression’ values on graph. The solutions and gel compositions are listed in Table S8.

### 2.22 Cerulein‐induced acute pancreatitis in guinea pig

Guinea pigs were exposed to cigarette smoke for 6 weeks as described above, and after the treatment period acute pancreatitis (AP) was induced by 10 hourly injections of cerulein (50 µg/kg, i.p.) (Bachem AG). Cerulein is a CCK‐analogue hormone that induces in the applied dose in the hypersecretion of the pancreatic enzymes. Control animals received physiological saline (PS). The guinea pigs were sacrificed 12 h after the first cerulein injection by terminal pentobarbital (85 mg/kg, i.p.) anaesthesia. To determine the serum amylase activity, blood was collected from the heart. All blood samples were centrifuged at 2500 *g* for 15 min and the serum was stored at −20°C. The pancreas was quickly removed, cleaned from fat and lymph nodes, frozen in liquid nitrogen and stored at −80°C until use. For histological analyses, pancreata were placed in 4% formaldehyde, paraffin‐embedded and 4 µm thick sections were cut for haematoxylin–eosin staining. To estimate severity of AP, oedema, inflammatory cell infiltration and necrosis were scored by three independent investigators who were blinded to the applied treatment (0−5 points for oedema and leukocyte infiltration or % of total area for necrosis). Averages of the obtained scores of each sample were included in the manuscript. Serum amylase activity was measured using a colorimetric kinetic method (Diagnosticum) as described previously.^29^

### 2.23 Cerulein‐induced CP in mice

FVB/N mice were exposed to cigarette smoke for 6 weeks as described above for the guinea pigs, and in the last 2 weeks of the treatment period, CP was induced by five series of 8 hourly PS (control group) or 50 µg/bw kg cerulein injections every third day. Representative scheme of the experimental setup shows the induction of CP (Figure S1). Twenty‐four hours after the last cerulein injection, mice were anaesthetised with pentobarbital (85 mg/kg, i.p.) and sacrificed through exsanguination through the heart. To evaluate the severity of CP, the extent of fibrosis was quantified by using Crossmon's trichrome staining and pancreas weight/body weight ratio was determined.

### 2.24 Statistical analysis

GraphPad Prism software was used for statistical analysis. All data are expressed as the means ± SEMs. The Shapiro–Wilk normality test was employed. Both parametric (the unpaired *t*‐test or one‐way analysis of variance with Tukey's multiple comparisons test) and non‐parametric (the Mann–Whitney test and Kruskal–Wallis test) tests were used based on the normality of data distribution. *p* < .05 was considered statistically significant.

## 3 RESULTS

### 3.1 Both the expression and function of CFTR are decreased in smokers and in patients with CP

**FIGURE 1:**
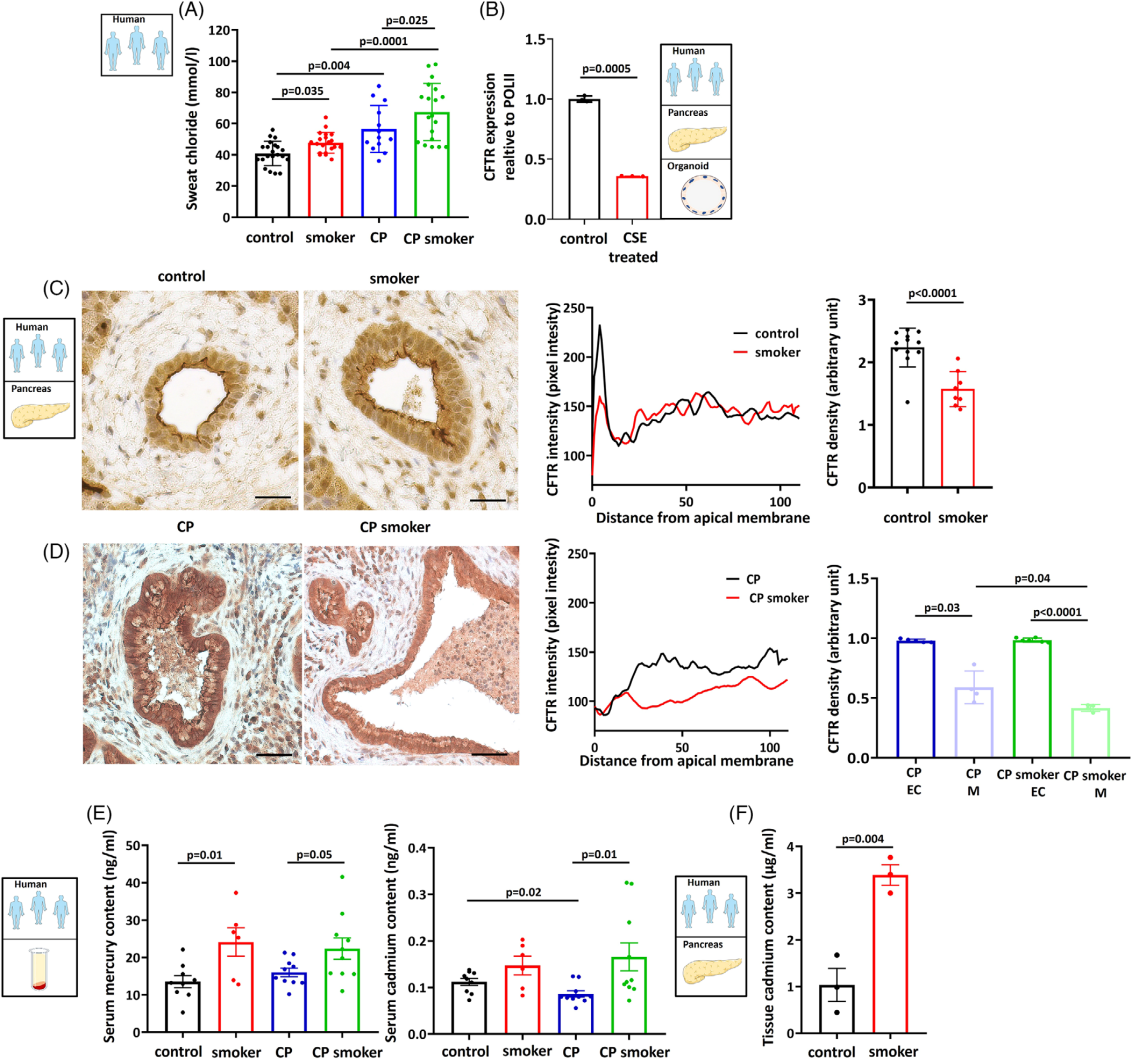
Cystic fibrosis transmembrane conductance regulator (CFTR) expression and activity are decreased in smokers and in chronic pancreatitis (CP) patients. (A) Cl_sw_^–^ was significantly higher in smoking patients (*n* = 16) compared to non‐smoking controls (*n* = 20). There is a significant difference in CP (*n* = 12) versus the control patients. Further elevation in sweat Cl^–^ concentration (Cl_sw_^–^) was observed in the smoking CP (*n* = 19) patients compared to the CP group. (B) Expression analysis showed that incubation of human pancreatic organoid cultures with 80 µg/mL cigarette smoke extract (CSE) significantly reduced the mRNA expression of CFTR. Expression levels were calculated relative to the POLII gene. (C) Representative confocal images showed that smoking significantly reduced CFTR expression on the apical membrane of pancreatic ductal epithelial cells in tissues from the human non‐smoker and smoker cadaver donors (*n* = 3). Scale bar = 50 µm. (D) The intensity profile confirmed that apical CFTR distribution was impaired in response to smoking. CFTR staining density at the luminal membrane was decreased in the smoking CP patients (H), whereas there was no significant difference in cytoplasmic density of CFTR between the non‐smoker CP (G) and smoker CP groups (*n* = 3). EC: cytoplasm; M: membrane. Scale bar = 50 µm. Exact *p*‐values are indicated above each column. (E) Analysis of the human serum samples showed that their mercury (Hg) content was significantly higher in smoker groups, both in the control and CP patients (*n* = 7−10). No significant difference in serum cadmium (Cd) concentration was observed between the non‐smoker (*n* = 9) and smoker (*n* = 7) controls. However, there was a significantly higher Cd concentration in the smoking CP (*n* = 10) versus non‐smoking CP (*n* = 10) patients. (F) Cd level was significantly higher in human pancreatic tissue samples derived from the smoker compared to the non‐smoker cadaver donors (*n* = 3).

A sweat Cl^–^ test, which is generally used to assess CFTR protein function in individuals suspected of having cystic fibrosis (CF), was conducted to compare CFTR function. In patients with CF, sweat Cl^–^ concentration (Cl_sw_^–^) is elevated due to diminished CFTR absorptive activity.^30^ Sweat samples were collected from the control individuals, smokers, and both smokers and non‐smokers with CP to compare CFTR function. In the control non‐smokers, Cl_sw_^–^ was 37.75 ± 1.48 mmol/L, whereas Cl_sw_^–^ was significantly elevated to 49.2 ± 1.66 mmol/L in the smokers without CP (Figure 1A). Elevation of C_sw_^–^ was also observed in the non‐smoking CP patients, which was even higher (54.25 ± 3.8 mmol/L) than in the smokers. The highest elevation was detected in the smoker CP group (67.42 ± 4.2 mmol/L), which reached the level of Cl_sw_^–^ observed in the CF patients. These results suggested that smoking significantly elevates Cl_sw_^–^, which is correlated with a reduction in CFTR function, which is further deteriorated in CP patients (Figure 1A).

We established an *ex vivo* model using 3D human pancreas organoid culture treated with CSE to test whether smoking directly affects the mRNA expression of CFTR in the smokers (Figure 1B). Organoids were incubated with 80 µg/mL CSE for 24 h, which significantly impaired the mRNA expression of CFTR (Figure 1B). This finding was further confirmed in CSE‐treated CAPAN‐1 cells (Figure S2). Next, pancreatic tissue samples were collected from the non‐smoker and smoker controls (Figure 1C) and from non‐smoker and smoker CP cadaver donors (Figure 1D) and immunostaining was performed to determine the effect of smoking on CFTR localisation. The intensity profiles and summary bar charts of the densities (Figure 1C) show reduced CFTR expression on the apical membrane of the pancreatic ductal cells in the smokers compared to the non‐smoking patients without CP (Figure 1C). A similar decrease was observed in the patients with CP. CFTR expression on the membrane was significantly decreased in the smoker CP patients versus the non‐smoker CP group (Figure 1D). However, the cytoplasmic density of CFTR did not change between the non‐smoking and smoking patients with CP (Figure 1D).

Cigarette smoke is a heterogenous mixture of several thousands of different compounds, including nicotine and heavy metals.^31^ We analysed the levels of 16 different heavy metals in the human serum samples to further characterise the effects of smoking on the exocrine pancreas (Table S1). Serum Hg concentration was 13.52 ± 1.59 ng/mL in the non‐smoker group, whereas it was significantly elevated to 24.14 ± 3.8 ng/mL in the smoker group (Figure 1E). Similarly, the serum Hg concentration of the non‐smoking CP patients was 16.02 ± 1.13 ng/mL, which is significantly lower than the levels in the smoking CP patients (22.4 ± 2.87 ng/mL). In the case of Cd, no significant difference was observed between non‐smoker (.11 ± .007 ng/mL) and smoker (.14 ± .02 ng/mL) controls (Figure 1E). However, significantly higher serum Cd concentration was measured in the smoking CP patients (.16 ± .03 ng/mL) compared to the non‐smoker CP group (.08 ± .006 ng/mL). Based on these results, we assessed the Cd concentration in the human pancreatic tissue samples derived from the non‐smoker and smoker cadaver donors. Significantly higher concentrations of Cd were detected in a tissue from the smoking patients (3.38 ± .38 µg/mL) compared to the non‐smokers (1.06 ± .61 µg/mL) (Figure 1F). Tissue Cd levels were in the µg/mL range, suggesting the accumulation of Cd in pancreatic tissue.

### 3.2 Chronic whole‐body smoke exposure reduces pancreatic fluid and bicarbonate secretion and inhibits CFTR expression and function in isolated guinea pig PDEC

**FIGURE 2:**
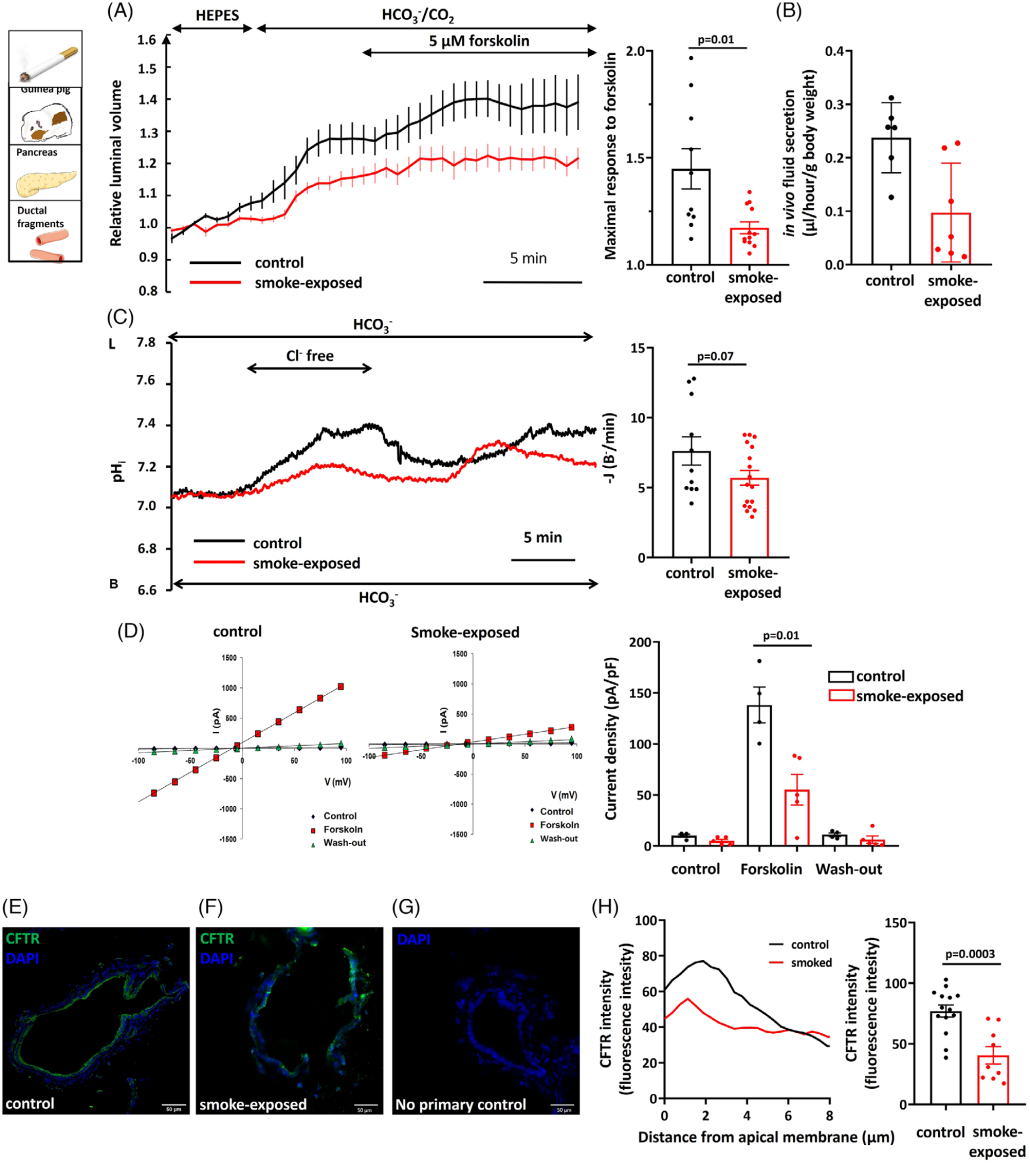
Smoke inhalation reduces pancreatic fluid and bicarbonate secretion and inhibits cystic fibrosis transmembrane conductance regulator (CFTR) Cl^–^ channel expression and function. (A and B) A six‐week period of smoke inhalation caused significantly reduced forskolin‐stimulated in vitro (A) and secretin‐stimulated in vivo (B) fluid secretion of isolated guinea pig pancreatic ducts. (C) Alkalisation of intracellular pH (pH_i_) caused by luminal Cl^–^ removal from the extracellular solution was significantly lower in isolated ducts from the smoke‐exposed animals (*n* = 17) compared to the control (*n* = 11), suggesting that HCO_3_^–^ secretion is reduced by smoking. L: luminal side; B: basal side. (D) The whole‐cell configuration of the patch clamp technique demonstrated that whole‐body smoke exposure significantly reduced the forskolin‐stimulated Cl^–^ current of CFTR in smoking guinea pig pancreas ductal epithelial cells (*n* = 4−5). (E–G) Confocal images indicated that the apical plasma membrane expression of CFTR was significantly decreased in guinea pig pancreatic ducts exposed to whole‐body smoke (F) compared to the control (E). (G) No‐primary antibody control. Green: CFTR; blue: DAPI. Scale bar = 50 µm. (H) Intensity profile of CFTR localisation confirmed that apical distribution was impaired because of the effect of smoking (*n* = 9−14). Exact *p*‐values are indicated above each column.

We exposed guinea pigs to whole‐body smoke for 6 weeks to assess the effect of smoking on the exocrine pancreas. First, pancreatic ductal fluid secretion was measured in vitro using isolated pancreatic ducts from smoke‐exposed and control guinea pig pancreata. The ducts were perfused with standard HEPES solution followed by standard HCO_3_^–^/CO_2_‐buffered solution, which induced an increase in the luminal volume. Ductal fluid secretion was stimulated with the cAMP agonist forskolin (5 µM), resulting in a further increase in luminal volume in the non‐smoking animals. In contrast, this rapid response was not observed in ducts isolated from the smoking guinea pigs (Figure 2A). To confirm these results, pancreatic juice was collected from anaesthetised mice in vivo. In response to secretin stimulation in vivo, ductal fluid secretion was significantly impaired by smoke inhalation in mice (Figure 2B). Next, pancreatic ductal HCO_3_^–^ secretion was measured with the luminal Cl^–^ withdrawal technique. Under these conditions, the ductal fragments isolated from the cigarette smoke‐exposed animals showed a significantly lower response to the intraluminal Cl^–^ removal compared to the control animals, suggesting that HCO_3_^–^ secretion is reduced by smoking (Figure 2C). As CFTR is one of the main contributors to ductal HCO_3_^–^ secretion, we then investigated whether the decrease in HCO_3_^–^ secretion could be partially attributed to the decrease in CFTR activity. We used the whole‐cell configuration of the patch clamp technique to assess the activity of CFTR in pancreatic ductal cells. Our experiments demonstrated that 5 µM forskolin stimulates CFTR Cl^–^ current in control PDEC not exposed to cigarette smoke (Figure 2D). However, this stimulatory effect was almost completely abolished in smoke‐exposed guinea pig PDEC. Moreover, the apical plasma membrane abundance of CFTR was significantly decreased in guinea pig pancreatic ducts exposed to whole‐body smoke compared to control ducts (Figure 2E–H). Overall, these results suggest that smoking decreases both the expression and activity of CFTR in PDEC and thus impairs pancreatic ductal ion and fluid secretion.

### 3.3 Cigarette smoke extract reduces pancreatic fluid and bicarbonate secretion and inhibits CFTR expression and function in pancreatic ductal fragments isolated from non‐smoking guinea pigs

**FIGURE 3:**
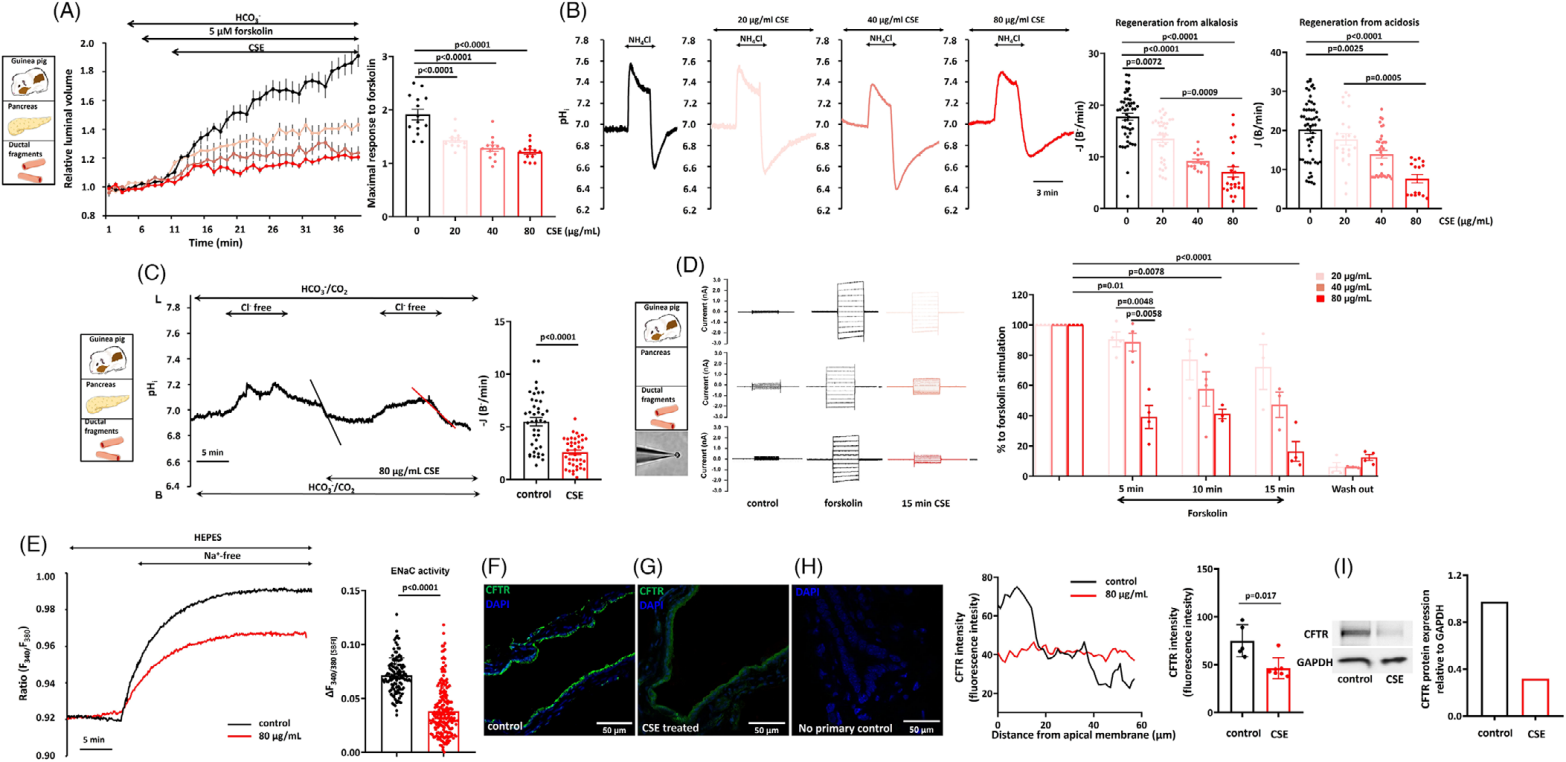
Cigarette smoke extract (CSE) reduces pancreatic fluid and bicarbonate secretion, inhibits cystic fibrosis transmembrane conductance regulator (CFTR) Cl^–^ channel expression and function and ENaC activity. (A) CSE treatment in 20, 40 and 80 µg/mL concentrations significantly inhibited the forskolin‐stimulated fluid secretion of pancreatic ducts isolated from the control guinea pigs. (B) Representative pH_i_ traces show that regeneration from alkalosis evoked by 20 mM NH_4_Cl was significantly and dose‐dependently reduced after 30 min CSE pretreatment. Recovery from acid load was also diminished by preincubation of the isolated pancreatic ducts with 20, 40 and 80 µg/mL CSE, suggesting that the activity of luminal and basolateral transporters may also be impaired. (C) The initial rate of pH_i_ recovery after luminal Cl^−^ re‐addition was significantly lower after preincubation of pancreatic ducts with 80 µg/mL CSE for 30 min (*n* = 5). (D) Patch clamp measurements showed that CSE significantly and irreversibly decreased the CFTR‐mediated forskolin‐stimulated, Cl^–^ current‐, time‐ and dose‐dependent manner in guinea pig pancreatic ductal epithelial cells (PDECs) (*n* = 4). (E) Representative traces and summary data of the Δ*F*_344/380_ show the effect of 80 µg/mL CSE on [Na^+^]_i_. ENaC activity was significantly decreased after CSE treatment. (F–H) Confocal images indicate that localisation of CFTR on apical plasma membrane is significantly decreased in guinea pig pancreatic ducts after pretreatment with 80 µg/mL CSE (G) compared to the control (F). (H) No‐primary antibody control. Scale bar = 50 µm. Green: CFTR; blue: DAPI. Intensity profile of CFTR localisation confirmed that apical distribution was impaired because of the effect of CSE preincubation (*n* = 5−7). Exact *p*‐values are indicated above each column. (I) Western blot analysis of CFTR protein level: protein samples were isolated from guinea pig pancreas organoid cultures after 12 h 80 µg/mL CSE incubation. CFTR protein level is markedly decreased in the pancreas after incubation for 12 h with 80 µg/ml CSE.

Smoke inhalation may have multiple indirect effects on exposed animals, so we developed an in vitro model to assess the direct effect of cigarette smoke on PDEC. In these experiments, pancreatic ducts isolated from control guinea pigs were incubated with different concentrations of CSE.^32^ First, we demonstrated that incubating PDEC with CSE has no effect on cell viability, as no apoptosis or necrosis was detected (Figure S3). Similar to whole‐body smoke exposure, CSE treatment inhibited the forskolin‐stimulated fluid secretion of isolated pancreatic ducts (Figure 3A). Pancreatic ductal HCO_3_^–^ secretion was measured with two complementary in vitro methods (NH_4_Cl pulse and luminal Cl^–^ removal). Isolated pancreatic ducts were exposed to 20 mM NH_4_Cl in HCO_3_^−^/CO_2_‐buffered solution from the basolateral membrane to assess the effect of CSE on HCO_3_^–^ secretion, which caused rapid alkalisation (Figure 3B). Similar to fluid secretion, CSE pretreatment significantly and concentration‐dependently diminished ductal HCO_3_^–^ secretion and inhibited the recovery rate from acid load, suggesting that the activity of basolateral transporters may also be impaired (Figure 3B). In addition, after the removal of luminal Cl^–^, the alkalisation of pH_i_ was significantly reduced by 80 µg/mL CSE perfusion compared to control ducts (Figure 3C). The patch clamp measurements confirmed that exposure of guinea pig PDECs to CSE significantly decreased the CFTR‐mediated forskolin‐stimulated Cl^–^ current, which was time‐ and concentration‐dependent and irreversible (Figure 3D). For the measurement of intracellular Na^+^ concentration ([Na^+^]_i_), cells were perfused with standard HEPES‐buffered solutions for 5 min, after the perfusion was switched to Na^+^‐free HEPES solution. Re‐addition of Na^+^‐containing HEPES solution caused elevation of [Na^+^]_i_, which was correlated with the activity of ENaC. If the cells were treated with 80 µg/mL CSE for 30 min, the maximal increase in [Na^+^]_i_ was significantly lower compared to untreated cells (Figure 3E). CFTR expression on the apical membrane of isolated pancreatic ductal fragments was significantly reduced after the CSE treatment (Figure 3G) compared to the control ducts (Figure 3F,H). Finally, to confirm the effect of CSE on the CFTR protein, we isolated total protein content of guinea pig pancreatic organoid cultures and performed western blot analysis for detection of CFTR. The results of this analysis further confirmed that the expression of CFTR in the pancreas was markedly decreased after incubation for 12 h with 80 µg/mL CSE (Figure 3I). Importantly, these results highlight that components of CSE directly impair the pancreatic ductal fluid and HCO_3_^–^ secretion as well as CFTR expression and activity.

### 3.4 Whole‐body smoke exposure and cigarette smoke extract reduce the maximal elevation of [Ca^2+^]_i_, ATP level and mitochondrial membrane potential in PDEC

**FIGURE 4:**
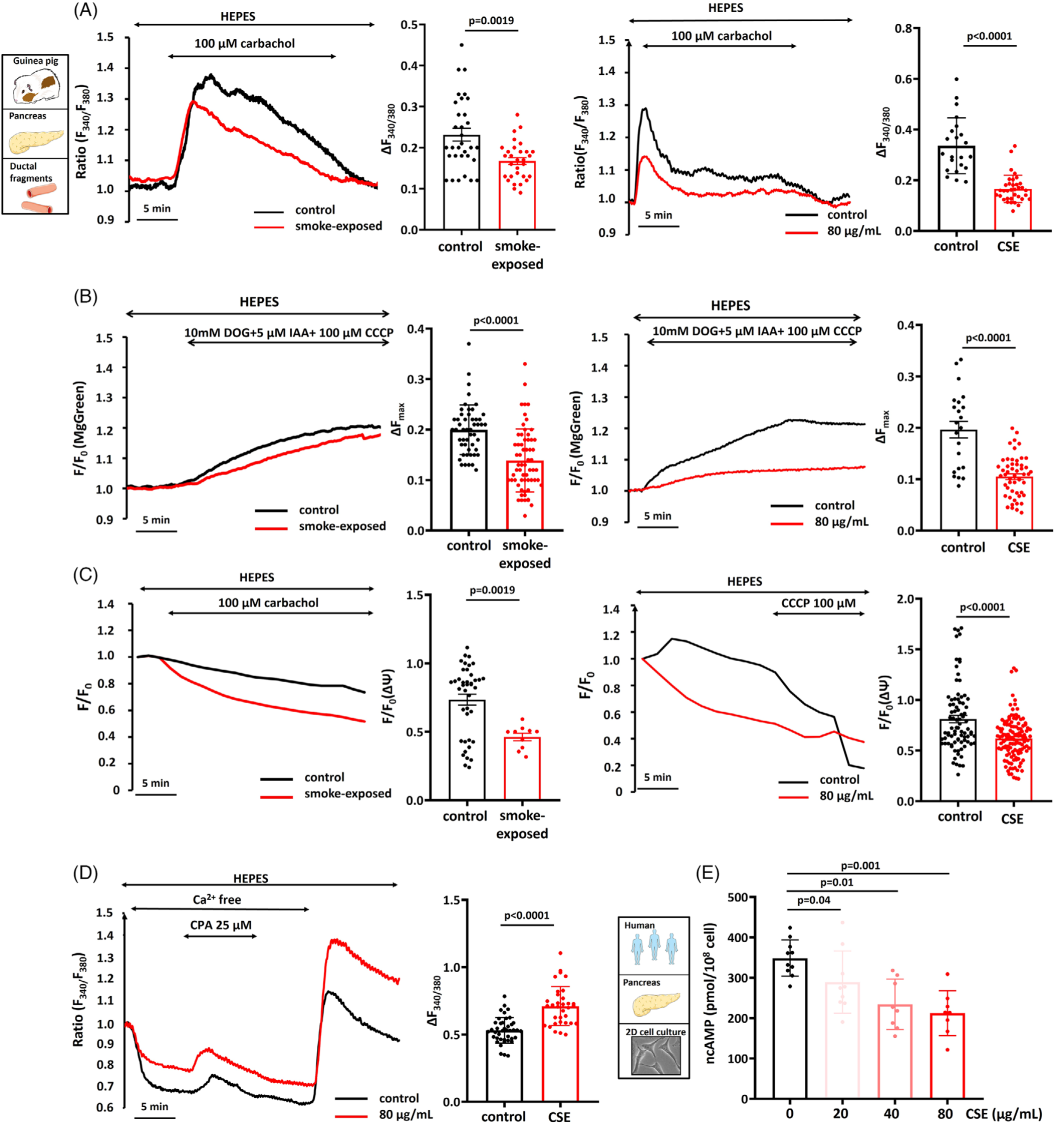
Smoke inhalation and cigarette smoke extract (CSE) reduce the maximum elevation of [Ca^2+^]_i_, ATP level and decrease mitochondrial membrane potential in isolated guinea pig pancreatic ductal cells. (A) Representative traces and summary data of the δRatio_max_ showing the effect of smoking and CSE on [Ca^2+^]_i_. The maximum response to 100 µM carbachol was significantly smaller in pancreatic ductal fragments isolated from the smoking animals and also after pretreatment with 80 µg/mL CSE (*n* = 5−7). (B) Total ATP depletion induced by a combination of deoxyglucose/iodoacetate/carbonyl cyanide 3‐chlorophenylhydrazone showed a significantly lower level of intracellular ATP in pancreatic ductal fragments isolated from the smoking animals or treated with CSE (*n* = 5−7). (C) Mitochondrial membrane potential was significantly diminished by smoking or CSE incubation (*n* = 5−7). (D) Representative traces of [Ca^2+^]_i_ demonstrating that CSE incubation significantly reduced extracellular Ca^2+^ influx in guinea pig pancreatic ductal fragments. (E) Summary data for cAMP measurements showing that total intracellular cAMP level was significantly reduced after preincubation of 20, 40 and 80 µg/mL CSE in CAPAN‐1 cells (*n* = 3).

We conducted intracellular Ca^2+^ and ATP measurements and assessed mitochondrial integrity to evaluate the molecular mechanism underlying the impact of smoke on ductal HCO_3_^–^ secretion and CFTR activity. We used pancreatic ductal fragments isolated from the non‐smoking and smoking guinea pigs in parallel with ductal fragments treated with CSE. The maximum elevation of [Ca^2+^]_i_ induced by 100 µM carbachol was significantly smaller in PDEC isolated from a smoke‐exposed animal and in ductal fragments treated with CSE (Figure 4A). We used a combination of deoxyglucose/iodoacetate/carbonyl cyanide 3‐chlorophenylhydrazone to assess the ATP_i_ levels and found that the total ATP depletion in the cell, which inhibits both glycolytic and mitochondrial ATP production, resulted in a significantly lower drop of ATP_i_ levels in pancreatic ductal fragments isolated from the smoke‐exposed animals or treated with CSE (Figure 4B). These results suggest that smoking impairs ATP_i_ production in the ductal cells. This was also suggested by measurements of the mitochondrial membrane potential, which was significantly decreased by smoking and CSE incubation (Figure 4C). Store‐operated Ca^2+^ entry (SOCE) was measured to further characterise the effect of CSE on Ca^2+^ signalling of pancreatic ducts isolated from the non‐smoking animals. In Ca^2+^‐free extracellular solution, 25 µM cyclopiazonic acid depleted ER Ca^2+^ stores and activated SOCE. Re‐addition of 1 mM extracellular Ca^2+^ evoked a marked extracellular Ca^2+^ influx, which was significantly higher after CSE treatment (Figure 4D).

A colorimetric competitive immunoassay kit was used on a human polarised pancreatic cell line (CAPAN‐1) to measure the effect of CSE administration for total intracellular cAMP concentration (Figure 4E). Similar to our other result, 30 min preincubation of CAPAN‐1 cells with different concentrations of CSE caused a significantly lower cAMP level compared to the control (Figure 4E).

### 3.5 Nicotine, mercury and cadmium inhibit the pancreatic ductal functions in PDEC isolated from non‐smoking animals

**FIGURE 5:**
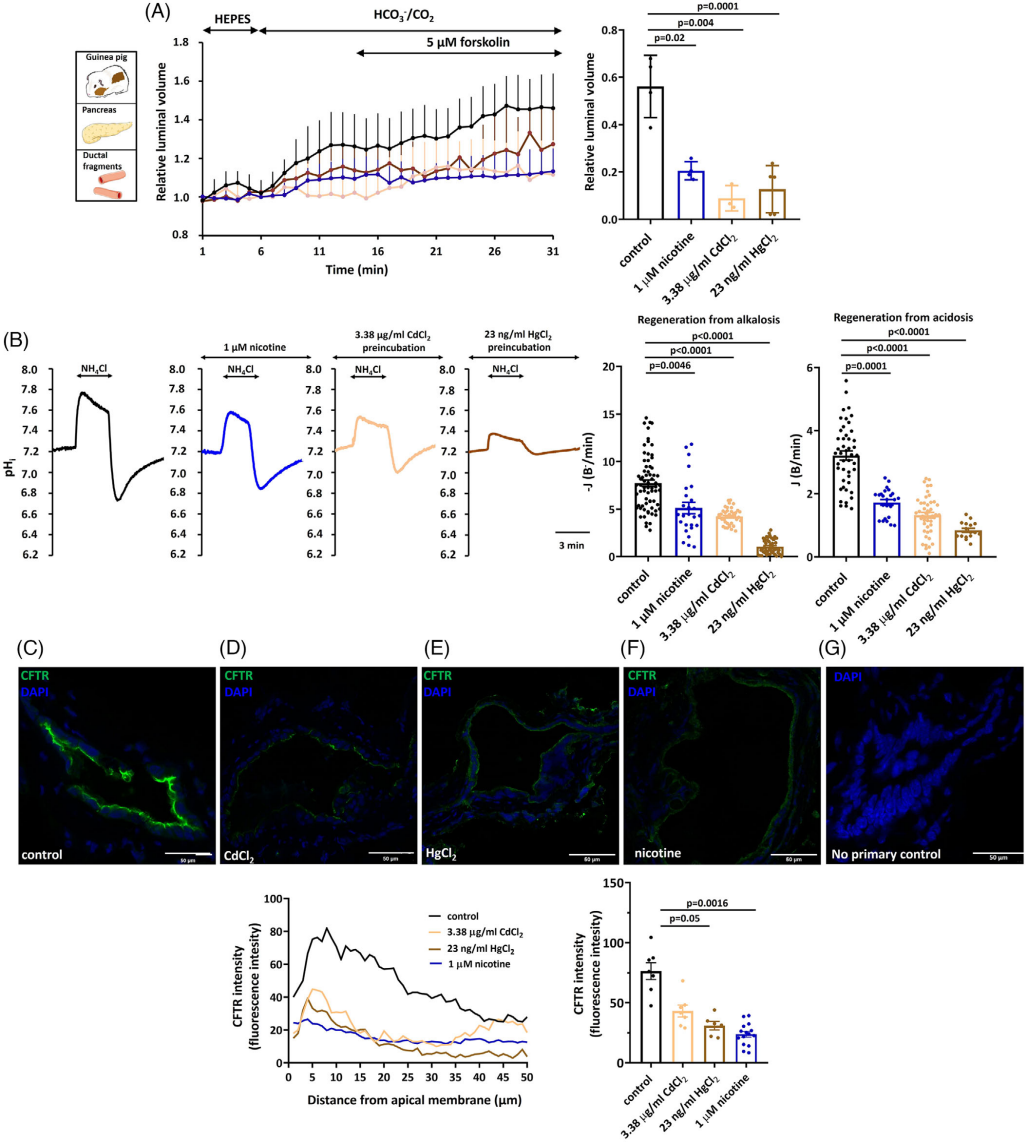
Nicotine, mercury and cadmium inhibit the functions of isolated guinea pig pancreatic epithelial cells. (A) Average traces and summary bar charts showing that forskolin‐stimulated fluid secretion into the closed luminal space was inhibited by administration of 1 µM nicotine (*n* = 4), 3.38 µg/mL Cd (*n* = 3) or 23 ng/mL Hg (*n* = 5) compared to the control. (B) Representative pH_i_ traces and summary bar charts demonstrating that regeneration from alkalosis and acidosis was significantly reduced by 30 min pretreatment with all three components of cigarette smoke. (C–F) Confocal images showing preincubation of isolated pancreatic ductal fragments with Cd (D), Hg (E) or nicotine (F) significantly reduced the apical localisation of cystic fibrosis transmembrane conductance regulator (CFTR). (G) No‐primary antibody control. Scale bar = 50 µm. Green: CFTR; blue: DAPI. An intensity profile of CFTR localisation confirmed that apical distribution was reduced after nicotine, Hg or Cd preincubation.

We performed the in vitro functional tests described above to test whether nicotine, Hg and the accumulated Cd in the pancreatic tissue of smokers can affect the pancreatic ductal functions. In these experiments, 30 min pretreatment of the control pancreatic ductal fragments with 1 µM nicotine, 23 ng/mL Hg (in a concentration that was equal to the serum levels in smokers) or 3.38 µg/mL Cd (at a concentration that was equal to the tissue levels in smokers) significantly impaired the pancreatic ductal fluid (Figure 5A) and HCO_3_^–^ secretion (Figure 5B). Moreover, all three components of cigarette smoke decreased the apical plasma membrane expression of CFTR (Figure 5C–G). These data suggest that nicotine, Cd and Hg inhibit the expression and function of CFTR on pancreatic ductal cells and may be responsible for the effect of smoking.

### 3.6 Smoking increases the severity of cerulein‐induced acute and chronic pancreatitis in guinea pigs and mice

**FIGURE 6:**
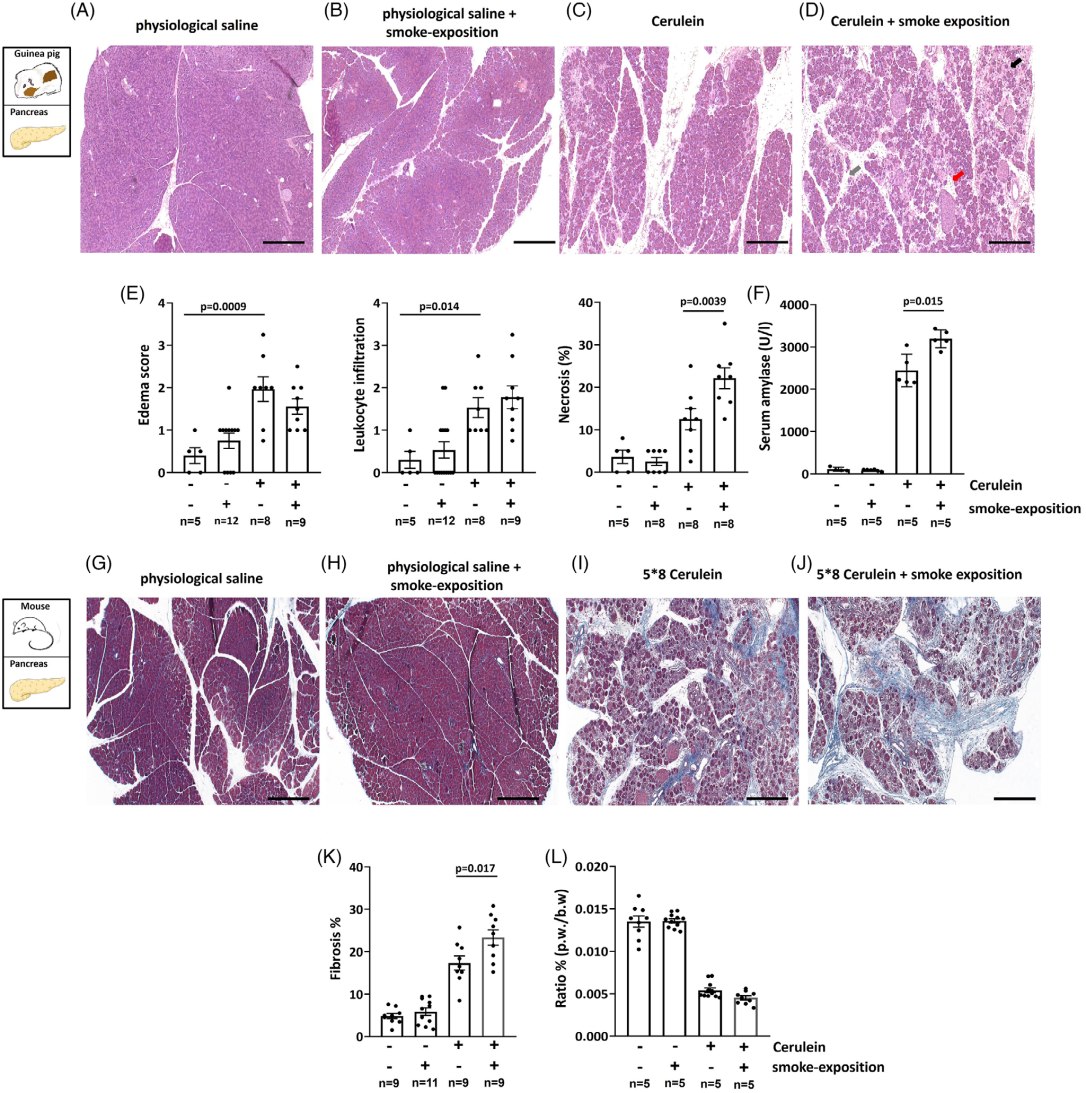
Smoking increases the severity of cerulein‐induced pancreatitis. Acute pancreatitis (AP) was induced by 10 h i.p. injections of either physiological saline (PS and control group) or 50 µg/kg cerulein after a 6‐week smoking period. (A–D) Representative haematoxylin and eosin (H&E) images of guinea pig pancreas. (A and B) PS and smoking alone caused no significant changes in pancreas histology. However, cerulein administration (C and D) induced a significant increase in pancreatic oedema (grey arrow on H&E image) (E), leukocyte infiltration scores (red arrow on H&E image) (E), necrosis (black arrow on H&E image) (E) or serum amylase activity (F). No significant differences were observed in the extent of interstitial oedema or leukocyte infiltration between the cerulein‐treated non‐smoker and smoker groups (E). However, the level of necrosis was significantly higher in the cerulein‐treated smoker group in comparison with the cerulein‐treated non‐smoking animals (E). Serum amylase activity was also significantly elevated in the cerulein‐treated smoking animals versus the cerulein‐treated non‐smoker group (F). Scale bar = 50 µm. Chronic pancreatitis (CP) was induced by repetitive i.p. injection of cerulein during the smoke‐exposed period in mice. In the last 2 weeks of the 6‐week smoking period used in previous experiments, the mice received five series of 8 hourly PS (control group) or 50 µg/bw kg cerulein injections every third day. (G–J) Representative Crossmon's trichrome staining images of mouse pancreas. Both the smoking and non‐smoking animals had normal pancreatic histology (G and H), whereas repetitive cerulein administration caused severe acinar cell atrophy, extensive fibrosis and the presence of acinar‐to‐ductal metaplasia (I and J), which was significantly worsened in the mice exposed to whole‐body smoke for 6 weeks (K). There was no significant difference was detected in pancreas weight/body weight ratio between the non‐smoker and smoker CP animals (L).

Experimental AP was induced with i.p. injections of cerulein to test whether smoking affects the severity of the disease. Both the smoking and non‐smoking animals had normal pancreatic histology (Figure 6A,B), whereas cerulein administration caused extensive pancreatic damage (Figure 6C,D). We observed no significant differences in the extent of interstitial oedema (grey arrow) or leukocyte infiltration (red arrow) between the cerulein‐treated non‐smoker and smoker groups (Figure 6E). However, the extent of necrosis (black arrow) was significantly higher in the cerulein‐treated smoker group compared to the cerulein‐treated non‐smoking animals (Figure 6E). Serum amylase activities were also significantly higher in the cerulein‐treated smoking animals versus non‐smoking animals (Figure 6F). Experimental CP was induced with repetitive i.p. injection of cerulein during the smoke‐exposed period in mice. In the last 2 weeks of the 6‐week smoking period used in previous experiments, the mice received five series of 8 hourly PS (control group) or 50 µg/bw kg cerulein injections every third day. Both the smoking and non‐smoking animals had normal pancreatic histology (Figure 6G,H), whereas repetitive cerulein administration caused severe acinar cell atrophy, extensive fibrosis and the presence of acinar‐to‐ductal metaplasia (Figure 6I,J), which was significantly worsened in the mice exposed to whole‐body smoke for 6 weeks (Figure 6K). There was no significant difference was detected in pancreas weight/body weight ratio between the non‐smoker and smoker CP animals (Figure 6L). To test the effect of 6‐week smoke inhalation on the lung, 11 parameters of lung function (frequency, tidal volume, minute ventilation, inspiratory time, expiratory time, tidal mid‐expiratory flow, peak inspiratory flow, peak expiratory flow, ratio of time to peak expiratory flow, functional residual capacity and airway resistance) were measured in mice with experimental CP. The results showed that the respiratory function parameters were not changed in any of the groups. All parameters are shown in Figure S4. We also measured the Cd content of lung after 6 weeks smoke exposure in mice. After the smoking period, a larger increase in the lung Cd content was observed in the smoked animals (∼4‐fold) than in the pancreas (∼2‐fold). Interestingly, in the non‐smoker animals, the Cd accumulation in the pancreas was significantly higher than in the lungs of the control animals suggesting that the dietary Cd intake is accumulated in the pancreas. The results are shown in Figure S5.

## 4 DISCUSSION

In this study, we demonstrated that cigarette smoking and CSE impair CFTR expression, activity and ductal secretory function in both human subjects and experimental models, resulting in more severe tissue damage in cerulein‐induced CP. Furthermore, our comprehensive analysis revealed that the concentrations of two heavy metals (Cd and Hg) were elevated in the serum samples of smokers and, importantly, Cd accumulated in the pancreatic tissue of smokers. These components of smoke, including nicotine, impaired ductal function and CFTR expression, thus suggesting that they play a crucial role in the development of cigarette smoke‐induced pancreatic ductal damage.

The ion and fluid secretion of epithelial cells is essential for the physiological function of several organs, such as the exocrine pancreas. This vectorial ion transport is largely determined by the activity of CFTR, which represents the rate‐limiting step for anion secretion.^33^, ^34^, ^35^ In contrast, the damaged expression and function of CFTR, as seen in CF^36^ or in acute^12^, ^37^ or CP^38^, ^39^ lead to pancreatic damage.^40^ It is also described that CFTR mutations are known risk factors of CP as suggested by the ‘ductal pathway of genetic risk’.^41^ Our group has previously demonstrated that the Cl^–^ concentrations are increased in sweat samples from patients acutely abusing alcohol but not in samples from healthy volunteers, indicating that alcohol affects CFTR function.^12^ Moreover, we also showed that pancreatic tissues from patients with acute or CP had lower levels of CFTR than tissues from healthy volunteers. In our current study, we observed that the Cl_sw_^–^ was significantly elevated in smokers compared to non‐smoker controls. Interestingly, the Cl_sw_^–^ was also significantly elevated in non‐smoking CP patients, whereas the highest elevation was observed in the smoker CP group, suggesting that the dysfunction of CFTR contributes to the CP pathogenesis. Therefore, it will be also interesting to investigate the combined effect of CFTR mutations and smoking on the development of CP.^41^ The treatment of human pancreatic organoids with CSE revealed that exposure of the ductal epithelial cells to cigarette smoke is sufficient to reduce the mRNA expression of CFTR. Furthermore, immunostaining of CFTR in human pancreatic tissue samples showed a pattern of CFTR expression similar to that suggested by the Cl_sw_^–^ measurements, as the CFTR expression on the luminal membrane of the ductal cells was impaired both in the smoking and non‐smoking CP patients, whereas the highest decrease was detected in the smoking CP patients. The effects of cigarette smoke on CFTR have been widely investigated in airway epithelial cells. An early elegant study by Cantin et al. showed that exposure of human airway cells to CSE decreases CFTR expression and function.^38^ Moreover, nasal potential difference was consistent in healthy smokers without CFTR mutations with subjects suffering from CFTR deficiency. Another study revealed that CSE inhibits the Cl^–^ secretion in human bronchial epithelial cells and increases mucin secretion.^32^ Importantly, the authors used human primary bronchial epithelial cells, which eventually increases the impact of their observations. Of note, e‐cigarettes were previously shown to inhibit the activity of CFTR and ENaC in airway epithelial cell.^42^ However, the effects of e‐cigarettes on the exocrine pancreas are currently unknown. Notably, our group showed previously that mucin production was also increased in CP in parallel with impaired pancreatic fluid secretion and CFTR function in mice and human tissues.^43^ In a well‐designed experimental work, Clunes et al. demonstrated that CSE exposure induces CFTR internalisation in airway epithelial cells, leading to air–surface liquid volume depletion and mucus dehydration.^44^

The effects of smoking and CSE exposure on ductal ion and fluid secretion were also investigated. Isolated ductal fragments from smoke‐exposed animals displayed significantly impaired in vivo and in vitro fluid secretion and apical Cl^–^/HCO_3_^–^ exchange activity compared to the controls. In addition, patch clamp measurements and immunostainings demonstrated a decreased CFTR activity and expression in smoked animals. The same alterations were found when ductal fragments were isolated from control animals and exposed to CSE in vitro, thus providing direct evidence that cigarette smoke is a triggering factor in ductal damage. When we assessed the intracellular mechanism underlying the inhibitory effect, we found impaired ENaC activity and [Ca^2+^]_i_ signalling and decreased ATP production and mitochondrial membrane potential, suggesting functional damage of mitochondria. These changes are very similar to effects described earlier for ethanol, bile acids and trypsin on PDEC.^12^, ^27^, ^45^

We also wanted to clarify which components of cigarette smoke damage ductal cells. Several studies have investigated the effects of CSE, which is a complex mixture of over 4000 compounds derived from the burning cigarette on sinonasal and bronchial epithelial cells. It has been previously demonstrated in human and mouse airway epithelial cells that CSE inhibited transepithelial Cl^–^ transport through CFTR^32^, ^46^ and the Ca^2+^‐activated Cl^–^ channel in airway epithelial cells.^47^ One dominant component is Cd, a toxic non‐essential transition metal, derived from cigarette smoking beside an agricultural or industrial source. The accumulation and harmful effect of Cd are well‐documented in several organs, such as the lungs, liver, kidneys and endocrine part of the pancreas.^48^ Hong et al. demonstrated that Cd exposure impairs pancreatic β‐cells function by disrupting the lipid metabolism, thus increasing the risk of diabetes.^49^ Another study showed that Cd exposure induces elevation of [Ca^2+^]_i_ and causes apoptosis of pancreatic β‐cells via c‐Jun N‐terminal kinase and C/EBP homologous protein‐related apoptotic signalling pathway.^50^ It has also been demonstrated that Cd increases reactive oxygen species production and causes oxidative stress, thus suppressing insulin secretion via apoptosis of pancreatic islet β‐cells.^51^ Of note the observations of these studies are somewhat questionable due to methodological limitations. The harmful effect of Cd on the endocrine pancreas is well documented. However, the role of Cd in the pathophysiology of the exocrine pancreas is unclear. In our study, we showed that both Cd and Hg concentrations are increased in the serum of human smokers. Moreover, Cd accumulated in the pancreatic tissue of smokers. This was also observed in the pancreas and lung of mice exposed to cigarette smoke for 6 weeks. When we analysed the effects of the components, we found that the incubation of PDEC with Cd, Hg or nicotine impairs the ductal ion and fluid secretion and reduces CFTR expression, suggesting that these components can mediate the harmful effects of cigarette smoke in the pancreas. Our findings on the effect of CSE and smoke exposure on CFTR activity align with other studies conducted on the lungs. A study has shown that exposure to Cd results in a reduction of CFTR protein expression, leading to decreased chloride transport in airway epithelia.^52^ Moreover, CFTR expression is also decreased in patients with severe COPD, and this effect is associated with the accumulation of Cd and manganese.^53^ A recent study highlighted that nicotine aerosols have been shown to reduce both airway CFTR function and mucociliary clearance.^54^

Overall, our findings demonstrated that (1) smoking and CSE diminish ductal fluid and HCO_3_^−^ secretion as well as the expression and function of CFTR, (2) Cd and Hg concentrations are significantly higher in the serum samples of smokers, and (3) Cd accumulates in the pancreatic tissue of smokers. Furthermore, we identified that the three major components of cigarette smoke, namely, nicotine, Cd and Hg, contribute to the detrimental effects of smoking in CP.

## AUTHOR CONTRIBUTIONS

The recommendations of the International Committee of Medical Journal Editors were followed to define authorship. The final order of the authors was determined based on the individual contributions of the authors. Péter Hegyi, Tamás Takács, Petra Pallagi and József Maléth designed the research project. Emese Tóth, Petra Pallagi, Marietta Görög, Viktória Venglovecz, Árpád Varga, Noémi Papp, Réka Molnár, Andrea Schnúr, Andrea Szentesi, László Czakó and Zoltán Rakonczay contributed to the acquisition, analysis and interpretation of the data for the study. Katalin Borka and Tamara Madácsy performed the histological investigations. Viktória Szabó and Enikő Kúthy‐Sutus prepared western blot analysis. Albert Kéri, Attila Ördög, Gyula Kajner and Gábor Galbács measured the heavy metal content of the serum and tissue samples. Cigarette smoke extract was performed by Dezső Csupor. Zsuzsanna Helyes and Kata Csekő conducted the smoke inhalation of the animals. Emese Ritter and Tünde Molnár performed chronic pancreatitis in mice. Petra Pallagi, József Maléth and Péter Hegyi drafted the article and all the authors approved the final version of the manuscript and agreed to be accountable for all aspects of the study in ensuring that questions related to the accuracy or integrity of any part of it were appropriately investigated and resolved. All persons designated as authors qualify for authorship, and all those who qualify for authorship are listed.

## CONFLICT OF INTEREST STATEMENT

The authors have no relevant financial or non‐financial interests to disclose.

## FUNDING INFORMATION

The authors received no specific funding for this work.

## ETHICS STATEMENT

The scheme of the experiments complied with the ethics of the research and was approved by the Medical Research Council of Hungary under ETT TUKEB 24169‐2/2018/EKU licence number. All experiments were performed in line with the Hungarian regulations and EU directive 2010/63/EU for the protection of animals used for scientific purposes and the study was approved by the National Scientific Ethical Committee on Animal Experimentation (licence no. XII./2222/2018).

## Supporting information

Supporting Information

## Data Availability

The datasets generated and/or analysed in the current study are fully available upon contact with the corresponding author.
