# Supplementary material for: Heavy metals in cigarette smoke strongly inhibit pancreatic ductal function and promote development of chronic pancreatitis
Source: Clin Transl Med. 2024 Jun 14;14(6):e1733. doi: 10.1002/ctm2.1733 (PMC11178517; doi:10.1002/ctm2.1733)
Supplement: Supplementary file 1 — Supporting Information [file CTM2-14-e1733-s001.docx]

**SUPPLEMENTARY FIGURES AND TABLES**


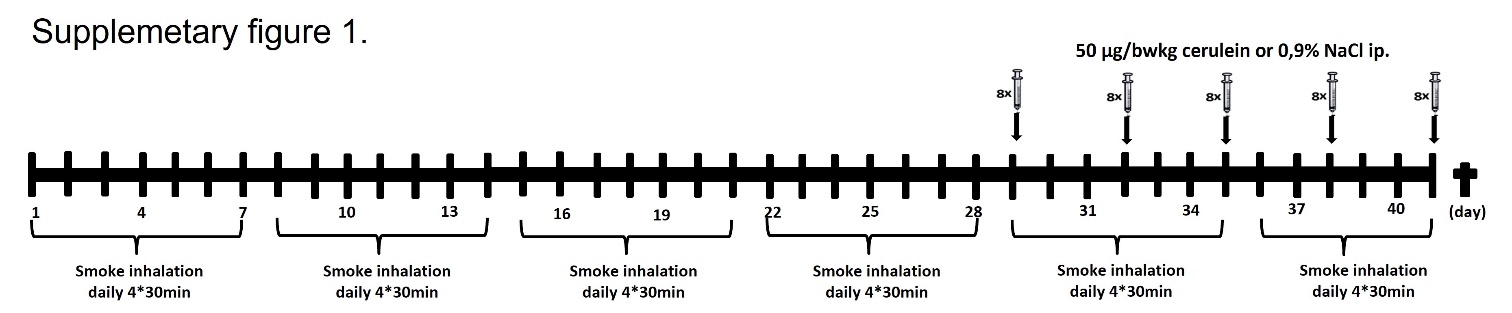


**Supplementary Figure 1.** Representative scheme of the experimental setup showing the induction of chronic pancreatitis (5 series of 8 hourly intraperitoneal cerulein injections every third day, 50 µg/bwkg) in mice during the 6-weeks smoking period.


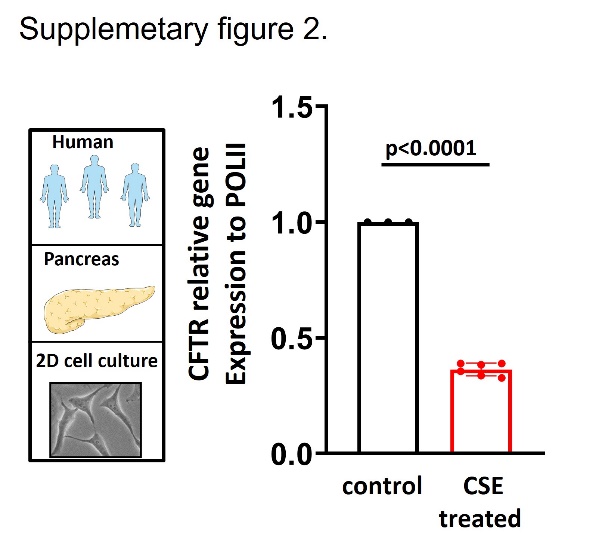


**Supplementary Figure 2. CFTR expression is decreased in CAPAN-1 cell line.** Expression analysis showed that 80 µg/ml CSE incubation was significantly reduced the mRNA level of CFTR in human CAPAN-1 cell culture.


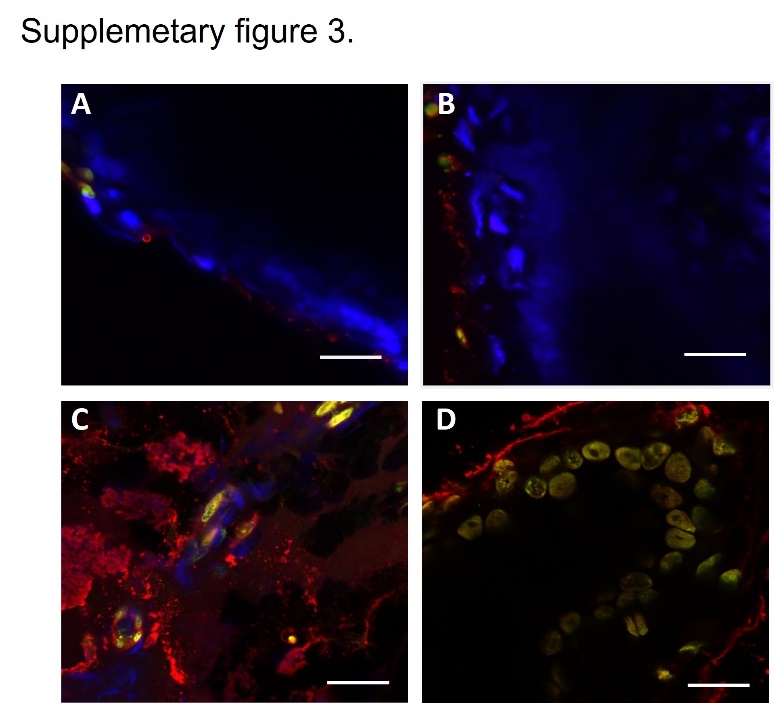


**Supplementary Figure 3. CSE incubation does not change the viability of pancreatic ductal cells.** Representative confocal *z*-stack images of pancreatic ductal fragments isolated from guinea pig under different treatment conditions (blue: live cells labelled with CytoCalcein 450, green: necrotic cells labelled with Nuclear Green, and red: apoptotic cells labelled with Apopxin Deep Red). A, non-treated pancreatic ducts. B-D, Pancreatic ducts incubated with 80 µg/ml CSE for 1 hour (B), with 100 µM CCCP (C) for induction of apoptosis, or 37 m/m % HCl (D) for induction of necrosis. Incubation of pancreatic ductal fragments with CSE has no effect on the cell viability as no apoptosis or necrosis was detected. Scale bar: 50 μm incubation.


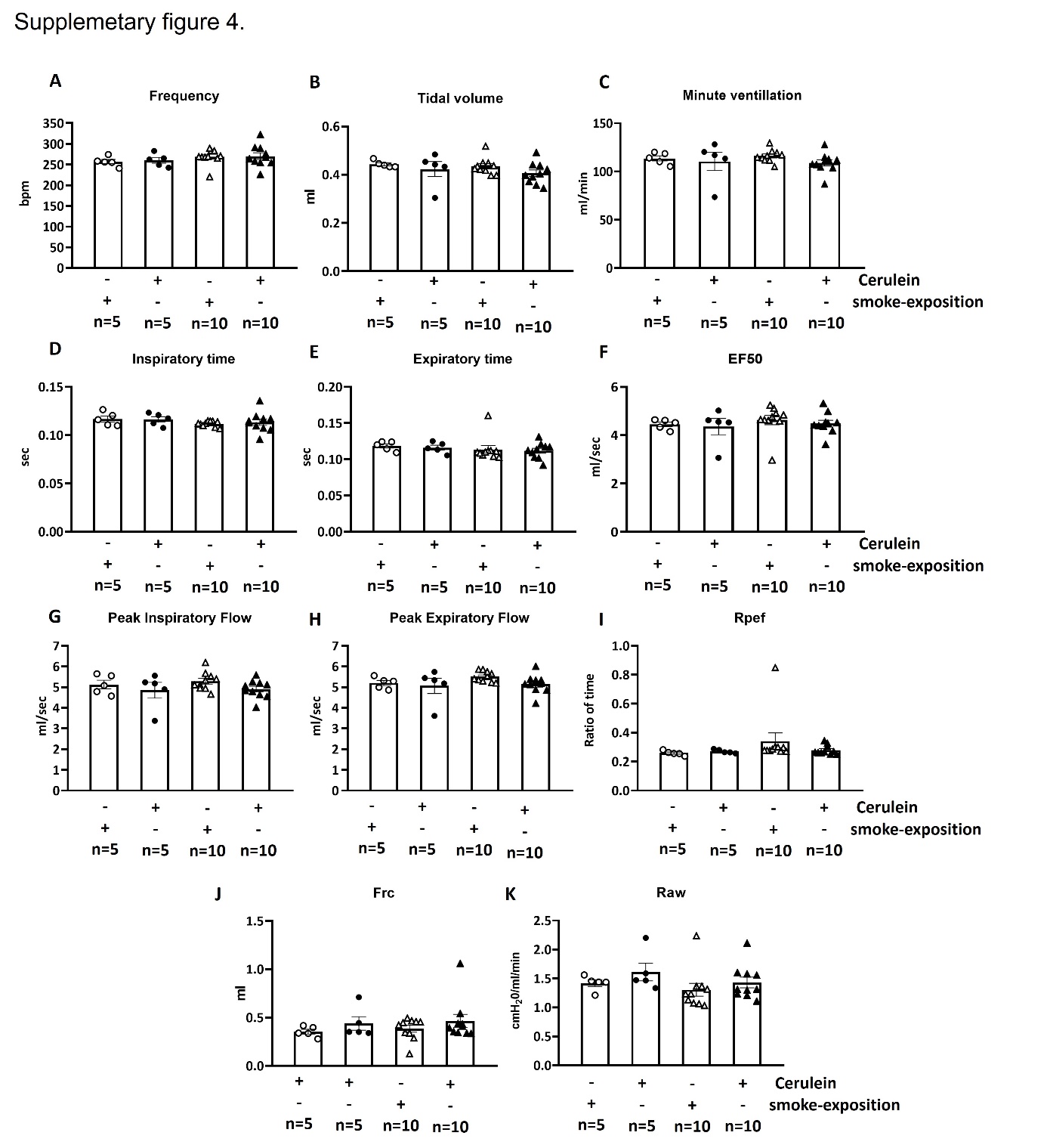


**Supplementary Figure 4. Respiratory function parameters were not changed in mice with chronic pancreatitis after 6-week smoke inhalation.** Frequency (**A**), tidal volume (**B**), minute ventilation (**C**), inspiratory time (**D**), expiratory time (**E**), tidal mid-expiratory flow (EF50) (**F**), peak inspiratory flow (**G**), peak expiratory flow (PEF) (**H**), ratio of time to peak expiratory follow (Rpef) (**I**), functional residual capacity (Frc) (**J**) and airway resistance (Raw) (**K**). n = 5-10/group.


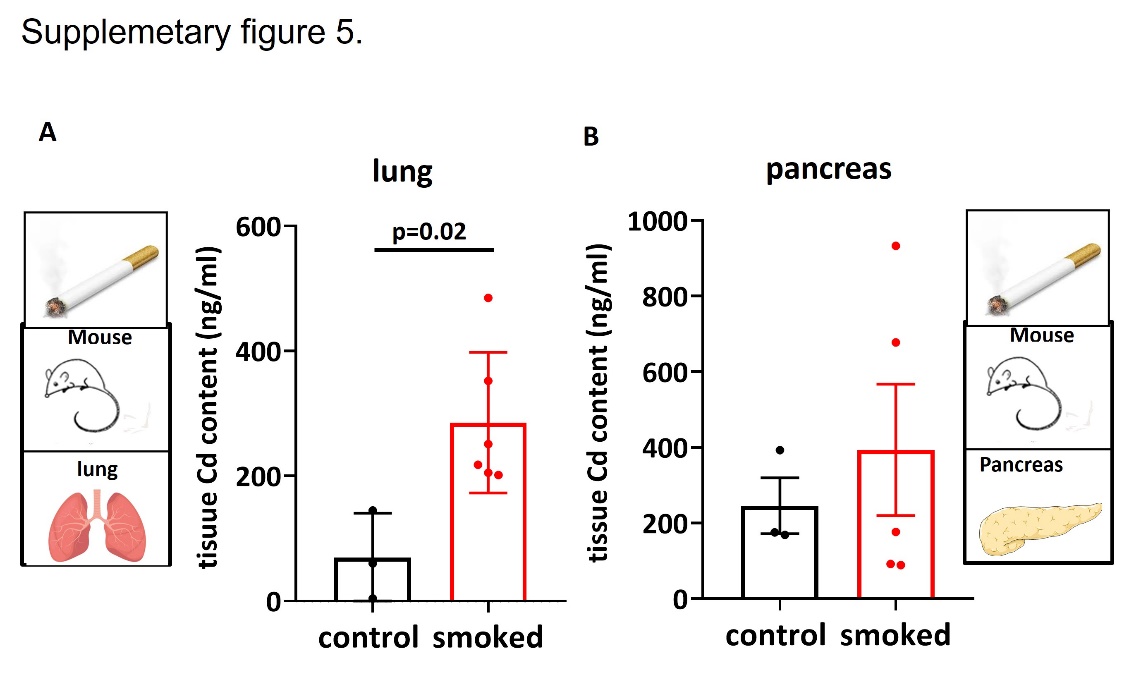


**Supplementary Figure 5.** Cd content of lung after 6 weeks smoke exposure in mice was significantly increased compared to non-smoker animals. After the smoking period, a larger increase of the lung Cd content was observed in the smoked animals (~4-fold) (**A**), compared to the pancreas (~2-fold) (**B**). n= 3-6 animal.

**Supplementary Table 1.** Heavy metal content of serum samples from patients

| heavy metals (ng/ml) | non-smoker | smoker | non-smoker with CP | smoker with CP |
| --- | --- | --- | --- | --- |
| B | 28.17889 | 48.735 | 42.75745 | 48.73356 |
| Al | 38.147 | 201.186 | 208.854 | 248.1978 |
| Cr | 84.04778 | 98.76667 | 68.77455 | 69.412 |
| Mn | 8.894333 | 12.65367 | 21.712 | 21.8861 |
| Fe µg/ml | 2.535889 | 3.369833 | 3.562091 | 3.1886 |
| Co | 2.242444 | 3.309333 | 2.823818 | 4.2421 |
| Ni | 7.038 | 21.01467 | 6.321091 | 10.4592 |
| Cu µg/ml | 1.095778 | 1.566833 | 1.373182 | 1.343 |
| Zn µg/ml | 1.194111 | 1.424833 | 1.346818 | 1.3195 |
| As | 11.58011 | 15.65833 | 12.68255 | 9.6054 |
| Se | 130.6889 | 169.2167 | 105.4445 | 81.915 |
| Mo | 1.425111 | 1.095667 | 2.350273 | 3.0311 |
| Cd | 0.112111 | 0.147333 | 0.0862 | 0.1659 |
| Sn | 8.148889 | 10.13383 | 6.334727 | 8.7399 |
| Hg | 13.52678 | 24.145 | 16.31 | 22.401 |
| Pb | 23.63163 | 34.68 | 18.73373 | 18.9295 |

**Supplementary Table 2** - Composition of solutions used during fluorescent measurements

|  | Standard HEPES | Ca^2+^-free HEPES | Standard HCO_3_^-^ | NH_4_Cl-HCO_3_^-^ | Cl^-^-Free HCO_3_^-^ |
| --- | --- | --- | --- | --- | --- |
| NaCl | 131313000 | 132 | 115 | 95 |  |
| KCl | 5 | 5 | 5 | 5 |  |
| MgCl_2_ | 1 | 1 | 1 | 1 |  |
| CaCl_2_ | 1 |  | 1 | 1 |  |
| Hepes | 10 | 10 |  |  |  |
| Glucose | 10 | 10 | 10 | 10 | 10 |
| NaHCO_3_^-^ |  |  | 25 | 25 | 25 |
| NH_4_Cl |  |  |  | 20 |  |
| Na-gluconate |  |  |  |  | 115 |
| K_2_-sulphate |  |  |  |  | 2.5 |
| Ca-gluconate |  |  |  |  | 6 |
| Mg-gluconate |  |  |  |  | 1 |

**Supplementary Table 3**. Splitting media.

| Component | Manufacturer/Cat.No. | Final cc/volume |
| --- | --- | --- |
| Advanced DMEM/F-12 | Gibco, 12634-010 | 500 ml |
| 1 M HEPES | Gibco, 15630080 | 5 ml  (10 mM) |
| GlutaMax Supplement (100X) | Gibco, 35050061 | 5ml  (1X) |
| Primocin  (400X) | Invivogen, ant-pm-2 | 1,25 ml  (1X) |

**Supplementary Table 4**. Digestion media.

| Component | Manufacturer/Cat.No. | Final cc/volume |
| --- | --- | --- |
| Splitting media | - | 20 ml |
| Collagenase IV. | Worthington, LS004188 | 1250 U/ml |
| Dispase | Sigma-Aldrich, D4693 | 0,5 U/ml |
| FBS | Gibco, 10500064 | 0,5 ml  2,5% v/v |
| Trypsin inhibitor | Sigma-Aldrich, T9128 | 1mg/ml |

**Supplementary Table 5**. Wash media.

| Component | Manufacturer/Cat.No. | Final cc/volume |
| --- | --- | --- |
| Splitting media | - | - |
| FBS | Gibco, 10500064 | 2,5% v/v |
| Antibiotic-Antimycotic Solution (100X) | Gibco, 15240062 | 1X |
| Kanamycin Sulfate (100X) | Gibco, 15160047 | 1X |
| Voriconazole | TOCRIS, 3760/10 | 2 µg/ml |

**Supplementary Table 6** – Feeding media of PSCs

| Component | Manufacturer/Cat.No. | Final cc/volume |
| --- | --- | --- |
| Dulbecco′s Modified Eagle′s Medium/Nutrient Mixture F-12 Ham | Sigma-Aldrich,  D6421 | 83 v/v% |
| Fetal Bovine Serum | Gibco, 10500064 | 15 v/v% |
| Antibiotic-Antimycotic Solution (100X) | Gibco, 15240062 | 1 v/v% |
| Kanamycin Sulfate (100X) | Gibco, 15160047 | 1 v/v% |
| Voriconazole | Sigma-Aldrich,V-032-1ML | 2 μg/ml |

**Supplementary Table 7.** Microwave-assisted digestion method used for the processing of samples

|  | Pancreatic tissue |
| --- | --- |
| Sample amount | 15-55 mg |
| Reagent mixtue | 3 mL HNO_3_, 1 mL H_2_O_2_) |
| Max. microwave power [W] | 1000 |
| Digestion temperature [°C] | 180 °C |
| Timing program | Ramp: 20 min, hold: 20 min |

**Supplemental Table 8 –** Solutions and gel compositions used in western blot

| 8% Running gel | | Stacking gel | |
| --- | --- | --- | --- |
| Name | Amount | Name | Amount |
| dH2O | 4.6 ml | dH2O | 2.975 ml |
| 30%Bis-acrylamide | 2.6 ml | 30% Bis-acrylamide | 0.67 ml |
| 1.5M Tris pH 8.8 | 2.6 ml | 0.5M Tris pH 6.8 | 1.25 ml |
| 10% SDS | 0.1 ml | 10% SDS | 0.05 ml |
| 10% APS | 0.1 ml | 10% APS | 0.05 ml |
| TEMED | 0.01 ml | TEMED | 0.005 ml |

| 5x Laemmli buffer (total of 10 ml) | |
| --- | --- |
| Name | Amount |
| 0.5M Tris pH 6.8 | 1.75 ml |
| Glycerol | 4.5 ml |
| SDS (0.25 g dissolved in 1 ml dH2O) | 2 ml 0.5 g total |
| 0.25% Bromophenol blue (25 mg in 10 ml dH2O) | 0.5 ml |
| 2- Mercaptoethanol | 1.25 ml |
